# Mutations in *MIR396e* and *MIR396f* increase grain size and modulate shoot architecture in rice

**DOI:** 10.1111/pbi.13214

**Published:** 2019-08-16

**Authors:** Chunbo Miao, Dong Wang, Reqing He, Shenkui Liu, Jian‐Kang Zhu

**Affiliations:** ^1^ State Key Laboratory of Subtropical Silviculture Zhejiang A&F University Lin'an Hangzhou China; ^2^ Key Laboratory of Molecular Biology and Gene Engineering in Jiangxi Province College of Life Science Nanchang University Jiangxi China; ^3^ Shanghai Center for Plant Stress Biology and Center for Excellence in Molecular Plant Sciences Chinese Academy of Sciences Shanghai China; ^4^ Department of Horticulture and Landscape Architecture Purdue University West Lafayette IN USA

**Keywords:** microRNA, rice, gene editing, grain size, plant architecture, gibberellin

## Abstract

Grain size and plant architecture are critical factors determining crop productivity. Here, we performed gene editing of the *MIR396* gene family in rice and found that *MIR396e* and *MIR396f* are two important regulators of grain size and plant architecture. *mir396ef* mutations can increase grain yield by increasing grain size. In addition, *mir396ef* mutations resulted in an altered plant architecture, with lengthened leaves but shortened internodes, especially the uppermost internode. Our research suggests that *mir396ef* mutations promote leaf elongation by increasing the level of a gibberellin (GA) precursor, mevalonic acid, which subsequently promotes GA biosynthesis. However, internode elongation in *mir396ef* mutants appears to be suppressed via reduced *CYP96B4* expression but not via the GA pathway. This research provides candidate gene‐editing targets to breed elite rice varieties.

## Introduction

The microRNA miR396, together with its targets the *Growth‐Regulating Factor* (*GRF*) genes, regulates many aspects of plant growth and development. miR396 negatively regulates plant cell proliferation (Debernardi *et al*., 2014; Liu *et al*., 2009; Rodriguez *et al*., 2009), and its overexpression in *Arabidopsis*, rice and tobacco led to severely retarded growth (Baucher *et al*., 2013; Gao *et al*., 2016a; Liu *et al*., 2009; Rodriguez *et al*., 2009; Tang *et al*., 2017). In *Arabidopsis thaliana*, the leaves show allometric growth with a specific growth polarity along the proximal‐distal axis, and miR396 controls this specific growth pattern via preferential accumulation in the distal part of young developing leaves (Rodriguez *et al*., 2009). A study surveying 75 eudicot species with different leaf growth polarities showed that the expression gradient of miR396 closely correlated with the polarity of leaf growth (Gupta and Nath, 2015), suggesting a conserved role for miR396 in leaf growth.

In rice, miR396 controls grain yield by regulating grain size and panicle architecture (Che *et al*., 2016; Duan *et al*., 2016; Gao *et al*., 2016a; Hu *et al*., 2015; Li *et al*., 2016). The rice grain size quantitative trait locus (QTL) *GS2*/*GL2* encodes GRF4 with a TC → AA transition at miR396‐targeting site and thus relieves its suppression by miR396, which further activates brassinosteroid (BR) response to increase the grain size (Che *et al*., 2016; Duan *et al*., 2016; Hu *et al*., 2015; Li *et al*., 2016). In addition, miR396 modulates panicle architecture through *GRF6* (Gao *et al*., 2016a). In rice, knockdown of miR396 greatly increased *GRF6* expression, which further activated auxin biosynthesis and response to generate larger panicles with more grains (Gao *et al*., 2016a). In tomato, decreasing miR396 expression enlarged flower organs and fruits (Cao *et al*., 2016). These findings suggest that negative regulation of fruit size is a general role for miR396.

Ideal plant architecture is critical for breeding elite crop varieties. In the past twenty years, plant architecture has been studied intensively, and some key regulators, including *SD1*,* OSDWARF4*,* DEP1* and *IPA1*, have been identified and used to improve rice productivity (Huang *et al*., 2009; Jiao *et al*., 2010; Miura *et al*., 2010; Sakamoto *et al*., 2005; Sasaki *et al*., 2002). Previous studies regarding plant architectures of monocot crops mainly focused on plant height, tillering, tiller angle, panicle architecture and leaf angle. The lengths of leaf blades and sheaths are also important aspects of plant architecture, but studies on this aspect are very limited in monocot crops.

Despite the known importance for miR396 as a key regulator of plant growth, its exact roles in plant growth and the underlying mechanisms are incompletely known. Here, through systematically mutating *MIR396* family genes in rice, we found that *MIR396e* and *MIR396f* are important regulators in grain size and plant architecture. *mir396ef* mutations resulted in greatly enlarged grains, lengthened leaf blades and sheaths, as well as shortened upper three internodes. Further analyses revealed that *mir396ef* promotes leaf elongation by increasing the level of a gibberellin (GA) precursor, mevalonic acid (MVA), which subsequently promotes GA biosynthesis. In addition, our results suggest that *mir396ef* mutations suppress internode elongation through decreasing *CYP96B4* expression. These findings provide candidate gene‐editing targets to breed elite rice varieties.

## Results

### *MIR396* gene editing in rice

**Figure 1:**
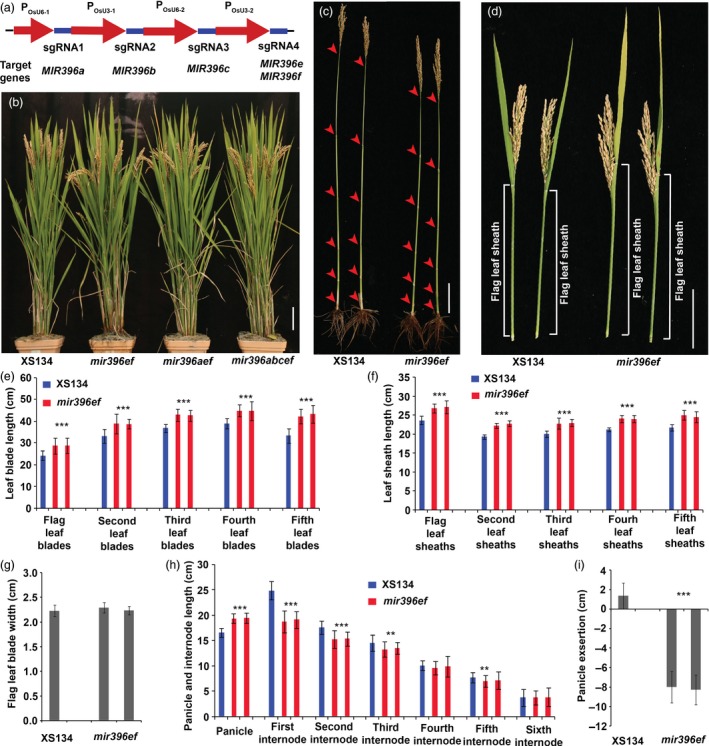
Shoot architecture analyses of the *mir396* mutants. (a) Strategy of vector construction for multiplex gene editing of *MIR396s*. Red arrows, promoters; violet‐blue boxes, sgRNA‐expressing sequences. (b) Wild‐type, *mir396ef*,* mir396aef* and *mir396abcef* plants at the mature stage. (c) Internode comparison between *mir396ef* and the wild type. Arrowheads, stem nodes. (d) Comparison of the flag leaf blades and sheaths between *mir396ef* and the wild type. (e) Leaf blade lengths of the wild type and *mir396ef* at the mature stage. (f) Leaf sheath lengths of the wild type and *mir396ef* at the mature stage. (g) Flag leaf blade widths of *mir396ef* and the wild type. (h) Lengths of panicles and internodes of wild‐type and *mir396ef* main tillers. (i) Quantification of the panicle exsertions from the flag leaf sheaths at the mature stage. Main tillers were used for the investigation. Each bar in the bar charts represents an independent line. Data are presented as means ± SD. *P* values (versus the wild type) were calculated with Student's *t*‐test. ***, *P *<* *0.001; **, *P *<* *0.01. Scale bars, 10 cm.

The rice genome contains eight *MIR396* genes (*MIR396a* to *MIR396h*) (http://www.mirbase.org/). Sequence analyses revealed that *MIR396a*,* MIR396b*,* MIR396c*,* MIR396e* and *MIR396f* locate to intergenic regions, whereas *MIR396d*,* MIR396g* and *MIR396h* locate to the exons of *GRF3*,* GRF2* and *GRF1*, respectively (Figure S1a). To dissect the function of the *MIR396* genes in rice, we constructed a multiplex CRISPR/Cas9 vector to simultaneously target *MIR396a*,* MIR396b*,* MIR396c*,* MIR396e* and *MIR396f* (Figure 1a; see Figure S1b for the target sites). We also constructed seven single sgRNA‐expressing vectors to mutate *MIR396a*–*MIR396d*,* MIR396g* and *MIR396h* individually, as well as *MIR396e* and *MIR396f* together (see Figure S1b for the target sites). The vectors were separately transformed into Xiushui 134 (XS134), an elite *japonica* cultivar widely cultivated by farmers in south‐east China. From T1 to T3 generations, we identified 174 homologous mutant lines for *mir396s*, including *mir396a*,* mir396b*,* mir396c*,* mir396d*,* mir396g*,* mir396h*,* mir396ab*,* mir396ef*,* mir396aef*,* mir396abef* and *mir396acef* (line A1–A174, Data S1). We also crossed *mir396abef* with *mir396acef* and identified 3 *mir396abcef* plants in F2 generation (line A175–A177, Data S1). In addition, we obtained *mir396e* and *mir396f* plants from the F2 generation of a cross between *mir396ef* and the wild type (line A178–A184, Data S1).

### *mir396ef* mutations increase leaf length but decrease the lengths of upper three internodes

Phenotypic analyses of the *mir396* mutants were conducted in the paddy fields of Hangzhou (China) and Hainan Island (China) under natural conditions. In the paddy field, the mutants including *mir396a*,* mir396b*,* mir396ab*,* mir396c*,* mir396d*,* mir396e*,* mir396f*,* mir396g* and non‐frame‐shift *mir396h* (*mir396h* with non‐frame‐shift mutations in *GRF1*) showed similar morphological phenotypes to the wild type during the entire life cycle. In contrast, the other mutants including *mir396ef*,* mir396aef*,* mir396abef*,* mir396acef* and *mir396abcef* showed apparent changes in shoot architecture when compared with the wild type (Figure 1b). At the mature stage in the paddy field, the panicles of *mir396ef* plants were obviously lower than those of the wild type, but its flag leaves were even higher than the wild type (Figure 1b). To explore this phenotypic difference in detail, we measured the lengths of the leaves and internodes at the seed‐filling stage. We found that all the leaf blades and sheaths in *mir396ef* were significantly longer than those in the wild type (Figure 1d–f), whereas no significant differences in the width of leaf blade were observed between *mir396ef* and the wild type (Figure 1g). Although the leaf length was remarkably increased, the upper three internodes in *mir396ef*, especially the uppermost internode (first internode), were significantly shorter than those in the wild type (Figure 1c, h), leading to a defect in panicle exsertion from the flag leaf sheath (Figure 1d, i). Thus, *mir396ef* mutations modulate shoot architecture by increasing the lengths of leaf blades and sheaths but decreasing the internode length. Higher‐order mutants, including *mir396aef*,* mir396abef*,* mir396acef* and *mir396abcef*, showed similar shoot architectures to *mir396ef* at the mature stage (Figure 1b).

*mir396ef* also displayed longer leaf blades and sheaths than the wild type during the seedling stage (Figure S2a–c). During this stage, *mir396ef* showed more robust growth than the wild type (Figure S2a). Higher‐order mutants, including *mir396aef*,* mir396abef*,* mir396acef* and *mir396abcef*, had similar morphological phenotypes to *mir396ef* during the seedling stage (Figure S2d). Neither miR396e nor miR396f (whose sequences differ from other miR396 members) was detected in *mir396ef* and *mir396abcef* seedling shoots by northern blot assays, consistent with complete disruption of *MIR396e* and *MIR396f* (Figure S3).

To further characterize the shortened internodes and lengthened leaves in *mir396ef*, we conducted histological analyses to compare the cell size between *mir396ef* and the wild type. Longitudinal sectioning analyses showed severely shortened cells in the uppermost internode of *mir396ef* (Figure S4a, b). Epidermal cells in the flag leaf blades and sheaths of *mir396ef* were slightly longer, but not wider, than those of the wild type (Figure S4c–h). Leaf cross sectioning revealed no obvious differences in cell size between *mir396ef* and the wild type (Figure S4i). These results indicate that cell elongation is slightly promoted in leaves but markedly suppressed in uppermost internode by *mir396ef* mutations.

Twelve *GRF* genes (*GRF1*–*GRF12*) have been identified in the rice genome, and among these twelve genes, eleven (*GRF1*–*GRF10* and *GRF12*) are targeted by miR396 (Choi *et al*., 2004; Kim and Tsukaya, 2015). Transcriptome analyses in the leaves (leaf blades and sheaths from 50‐day‐old plants) and developing uppermost internodes revealed that several miR396‐targeting *GRF* genes, especially *GRF3*, were up‐regulated by *mir396ef* mutations (Figure S5a, b). Among the miR396‐targeting *GRFs*,* GRF3* showed the highest expression level in both wild‐type and *mir396ef* leaves and developing uppermost internodes, and was up‐regulated by *mir396ef* mutations more intensely than most other *GRF* genes (Figure S5a, b), suggesting that *GRF3* is involved in the changed elongations of leaves and internodes in *mir396ef*.

### *mir396ef* mutations increase the sizes of grains and panicles

**Figure 2:**
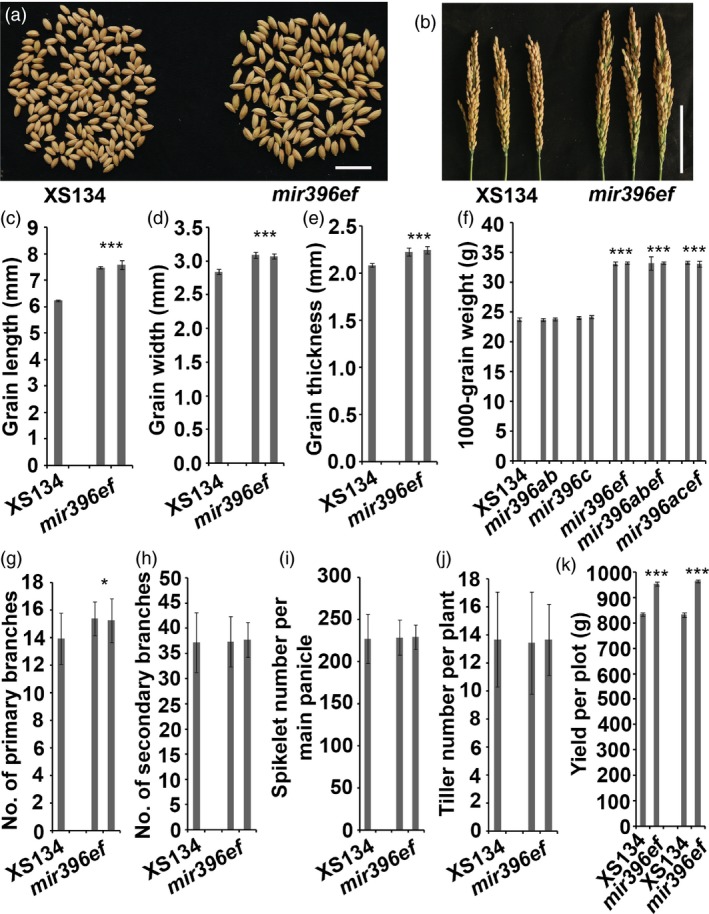
*mir396ef* mutations increased the size of grains and panicles. (a) Grains of *mir396ef* and the wild type. Scale bar, 2 cm. (b) Panicles of wild‐type and *mir396ef* main tillers. Scale bar, 10 cm. (c–e) Grain lengths (c), widths (d) and thicknesses (e) of *mir396ef* and the wild type. (f) 1000‐grain weights of *mir396ab*,* mir396c*,* mir396ef*,* mir396abef*,* mir396acef* and the wild type. (g and h) Numbers of primary (g) and secondary (h) branches per main panicle. (i) Spikelet numbers per main panicle of the wild type and *mir396ef*. (j) Tiller numbers of the wild type and *mir396ef*. (k) Grain yield per plot (90 × 60 cm) in Hainan. Two independent lines of every mutant were compared with the wild type. Data are presented as means ± SD. *P* values (versus the wild type) were calculated with Student's *t*‐test. ***, *P *<* *0.001; *, *P *<* *0.05.

The grains of *mir396ef* were obviously larger than those of the wild type (Figure 2a), and the grain length, width and thickness were significantly increased by *mir396ef* mutations (Figure 2c–e). The 1000‐grain weight was increased by about 40% by *mir396ef* mutations (Figure 2f). Both *mir396abef* and *mir396acef* seeds were similar in size to *mir396ef* seeds (Figure 2f). *mir396abcef* seeds were much larger than those of *mir396ef* (Figure S6a–e). However, the seed setting rate was much lower for *mir396abcef* than for *mir396ef* and the wild type (Figure S6f). As we did not observe obviously impaired fertility in *mir396a*,* mir396b*,* mir396c* and *mir396ab* when compared with the wild type, the severely decreased seed setting rate in *mir396abcef* suggests that the five *MIR396* genes (*MIR396a*,* MIR396b*,* MIR396c*,* MIR396e* and *MIR396f*) function redundantly in affecting fertility. Seeds of the single mutants (*mir396a* to *mir396h*) and *mir396ab* showed similar sizes to wild‐type seeds. Consistent with the enlarged grain size in *mir396ef*, scanning electron microscopy revealed that the epidermal cells of *mir396ef* spikelet hulls were longer and wider than those of the wild type (Figure S7c–e). Cross sectioning also showed that *mir396ef* spikelet hulls had obviously enlarged cells (Figure S7a, b).

The panicles of *mir396ef* were obviously longer and larger than the wild type (Figures 1h and 2b). Therefore, we compared the agronomic characteristics of the panicles between *mir396ef* and the wild type. We found that the primary branch number per main panicle in *mir396ef* was slightly larger than that in the wild type (Figure 2g), whereas the numbers of spikelets and secondary branches per main panicle were not significantly different between *mir396ef* and the wild type (Figure 2h, i). Therefore, the enlarged panicles in *mir396ef* accommodated enlarged grains rather than more grains. Higher‐order mutants, including *mir396aef*,* mir396abef*,* mir396acef* and *mir396abcef*, had similar panicle morphologies to *mir396ef*. Although enlarged grains and panicles were observed in *mir396ef*, tiller number per plant was not significantly affected by *mir396ef* mutations (Figure 2j).

The above results indicate that *MIR396e* and *MIR396f* are the main regulators of rice growth and development among the *MIR396* family genes. Next, using real‐time RT‐PCR, we analysed the expressions of *MIR396e* and *MIR396f* in 20‐day‐old seedlings, flag leaves, young panicles (about 3 cm), uppermost internodes and developing spikelets, and found that *MIR396e* and *MIR396f* were expressed most highly in young panicle and seedling shoot, respectively (Figure S8a, b).

### *mir396ef* mutations increase rice productivity

The above results suggest that *mir396ef* mutants could be a valuable resource for improving rice productivity. Therefore, we conducted paddy field plot yield test to evaluate the productivity of *mir396ef*. In Hainan, two independent *mir396ef* lines showed about 14% and 16% increases in grain yield, respectively (Figure 2k). However, we did not observe increased grain yields for *mir396ef* in Hangzhou (Figure S9a). Further investigation revealed bad seed filling and low fertility in the enclosed part of *mir396ef* panicles in Hangzhou (Figure S9b), suggesting that the panicle exsertion defect affected the seed filling and fertility in *mir396ef*.

### *mir396ef* mutations promote leaf elongation through the GA pathway

**Figure 3:**
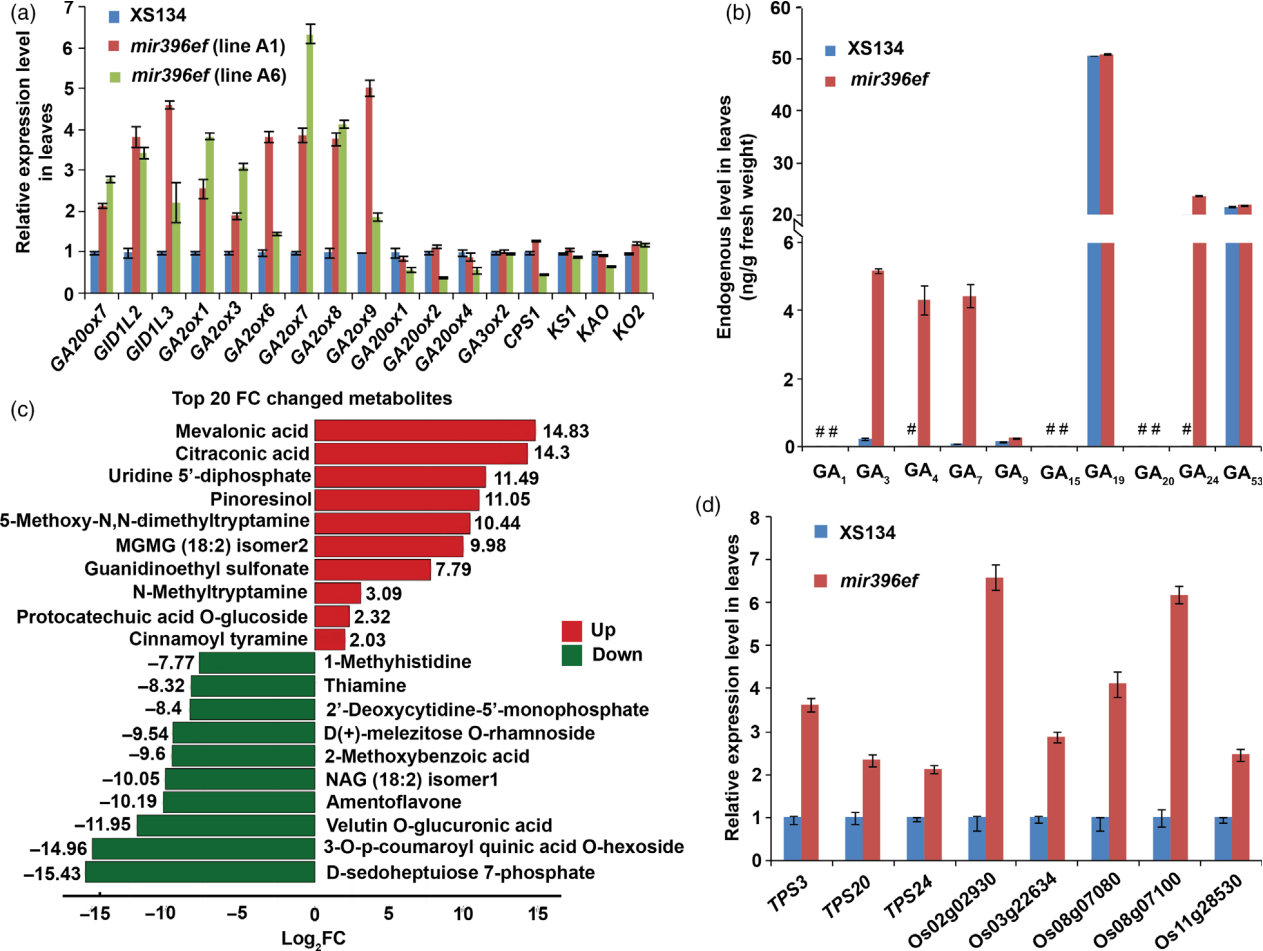
*mir396ef* mutations promote leaf elongation through the GA pathway. (a) Relative expression levels of GA‐related genes in *mir396ef* and wild‐type leaves. Two independent lines of *mir396ef* (line A1 and line A6) were used for the expression analyses. (b) Endogenous GA levels in *mir396ef* and wild‐type leaves. #, undetectable GAs. (c) The top 20 most markedly changed metabolites between *mir396ef* and wild‐type leaves. FC, fold change (*mir396ef*/XS134). (d) Relative expression levels of several *TPS* genes in *mir396ef* and wild‐type leaves. The leaves were sampled from 50‐day‐old plants in Hangzhou paddy field. Three independent biological replicates were performed, and error bars indicate standard deviation.

To explore the mechanism underlying the lengthened leaf blades and sheaths in *mir396ef*, we analysed the transcriptomes of leaves (including leaf blades and sheaths) from 50‐day‐old plants (Data S2 and S3). One thousand three hundred and eighty‐six differentially expressed genes (DEGs) were identified in the leaves of an *mir396ef* line (line A1) compared to the wild type (ratio ≥2 or ≤0.5, and false discovery rate (FDR) <0.05), including 873 up‐regulated and 513 down‐regulated genes (Data S4). Among the DEGs, a putative GA oxidase gene (LOC_Os08g44590, putative *GA20ox7*), two putative GA receptor genes (designated *GID1L2* and *GID1L3* here) and four GA‐deactivating genes (four *gibberellin 2‐oxidase* family genes including *GA2ox6, GA2ox7, GA2ox8* and *GA2ox9*; Lo *et al*., 2008) were markedly up‐regulated in *mir396ef* (Data S4 and S5). We also compared the expression profiles of all the detected GA biosynthetic, signalling, deactivating and response genes between wild‐type and *mir396ef* (line A1) leaves, and found that most GA‐deactivating and response genes were up‐regulated by *mir396ef* mutations (Data S5 and Figure S10). Transcriptome analysis with another independent *mir396ef* line (line A6) was also conducted, and the GA‐related DEGs (ratio ≥2 or ≤0.5, and FDR <0.05), including *GID1L2*,* GID1L3*, putative *GA20ox7*,* GA2ox1*,* GA2ox3*,* GA2ox7* and *GA2ox8*, also showed markedly increased expression in line A6 *mir396ef* leaves compared to the wild type (Data S6 and S7). We further confirmed these findings using real‐time RT‐PCR (Figure 3a).

Next, we measured GA levels in the leaves of 50‐day‐old plants through a high‐performance liquid chromatography‐mass spectrometry (HPLC‐MS) method. In contrast to the increased expression of the GA‐deactivating *GA2ox* genes in *mir396ef* leaves, much higher levels of the bioactive GAs including GA_3_, GA_4_ and GA_7_, as well as a bioactive GA precursor GA_24_, were observed in *mir396ef* compared with the wild type (*P *<* *0.001; Figure 3b). Thus, we speculated that the increased expression of *GA2ox* genes in *mir396ef* leaves may be a response to the increased GA levels. One of the most significant roles of GAs in plants is to promote organ elongation (Rizza *et al*., 2017). Therefore, the above results suggest that *mir396ef* mutations promote leaf elongation by increasing GA levels.

To explain the increased GA levels, we first examined the expression of the key genes in GA biosynthetic pathway, including *GA20oxs* (*GA20ox1*,* GA20ox2*,* GA20ox3* and *GA20ox4*), *GA3oxs* (*GA3ox1* and *GA3ox2*), *CPS1*,* KS1*,* KO2* and *KAO*. In GA biosynthetic pathway, GA20oxs and GA3oxs catalyse multiple late steps, whereas CPS1, KS1, KO2 and KAO catalyse four early steps (Sakamoto *et al*., 2004). The expression of *GA3ox1* and *GA20ox3* was not detected in the leaves of 50‐day‐old wild‐type and *mir396ef* plants, and the expression of other key GA biosynthetic genes did not show substantial differences between *mir396ef* and the wild type in both transcriptome and real‐time RT‐PCR analyses (Figure 3a, Data S5 and S7). The results suggest that the increased GA levels in *mir396ef* leaves do not arise from altered expression of GA biosynthetic genes.

Next, the leaf metabolome of 50‐day‐old *mir396ef* was compared with the wild type using a HPLC‐MS method which could detect 609 metabolites (Data S8). Through this assay, we detected 37 up‐regulated and 26 down‐regulated metabolites in *mir396ef* compared to the wild type (fold change (FC) ≥2 or ≤0.5, and variable importance in projection (VIP) ≥1; Data S9). Interestingly, among the 37 up‐regulated metabolites, mevalonic acid (MVA) showed the highest increased level in *mir396ef* mutants, increasing by about 29 000‐fold (log_2_FC = 14.83; FC, *mir396ef*/XS134) (Figure 3c). MVA is a precursor of the terpenoids, which constitute a large class of naturally occurring compounds in plants, including GAs and abscisic acid (ABA; Okada, 2011; Ruiz‐Sola *et al*., 2016). *Terpene Synthase* (*TPS*) family genes are responsible for the syntheses of many kinds of terpenoids (Falara *et al*., 2011). Consistent with the increased MVA level, the above transcriptome analyses also revealed significantly increased expression of *TPS*s in *mir396ef* leaves (Data S4 and S6). Among the DEGs (ratio ≥2 or ≤0.5, and FDR <0.05) identified in the above transcriptome analyses, 14 and 11 *TPSs* were found to be markedly up‐regulated in line A1 and line A6 *mir396ef* leaves, respectively (Table S1). Real‐time RT‐PCR confirmed the increased expression of the *TPSs* in *mir396ef* leaves (Figure 3d). The up‐regulation of *TPS* genes in *mir396ef* leaves may be a response to the increased MVA level.

In plants, MVA is converted to isopentenyl diphosphate (IPP) and dimethylallyl diphosphate (DMAPP), and then, IPP and DMAPP are used as common precursors of all terpenoid compounds including GAs and ABA (Figure S11; Okada, 2011; Ruiz‐Sola *et al*., 2016). Plants also utilize DMAPP as one precursor of cytokinins (CKs) (Figure S11; Ruiz‐Sola *et al*., 2016). Therefore, we also measured the levels of other phytohormones, including ABA and CKs, in the leaves of 50‐day‐old plants. Only slightly increased levels of ABA, N_6_‐isopentenyladenine (IP) and trans‐zeatin (tZ) were observed in *mir396ef*, and the levels of other CKs including cis‐zeatin (cZ) and dihydrozeatin (DZ) were not significantly increased in *mir396ef* mutant (Figure S12).

The above results suggest that *miR396ef* mutations promote leaf elongation by markedly increasing the MVA level, which subsequently promotes GA synthesis to activate the GA pathway.

### The inhibited internode elongation in *mir396ef* mutants is correlated with reduced expression of *CYP96B4*

**Figure 4:**
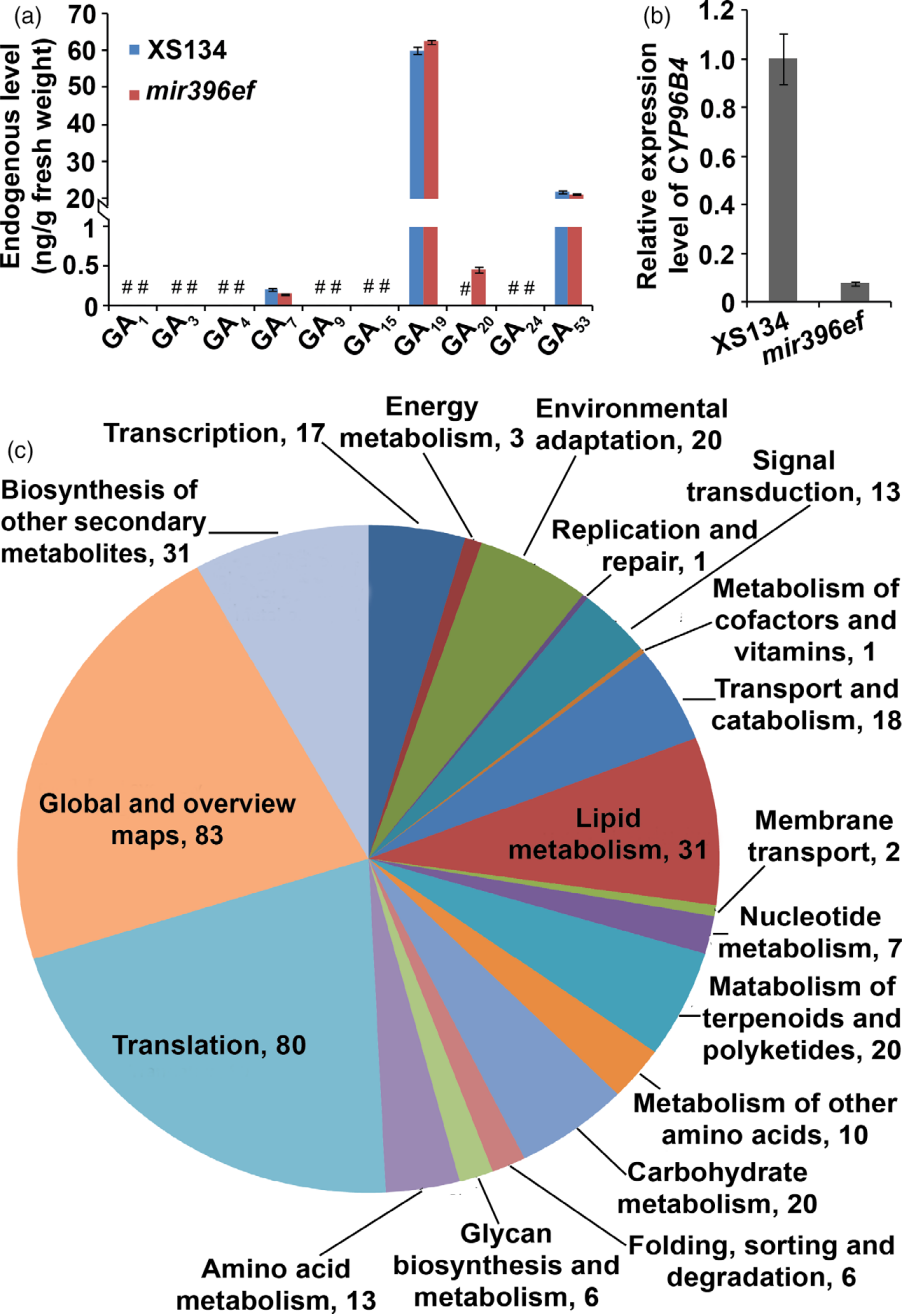
*mir396ef* mutations suppress internode elongation through reducing *CYP96B4* expression but not through the GA pathway. (a) Endogenous GA levels in developing uppermost internodes. #, undetectable GAs. (b) Relative expression level of *CYP96B4* in developing uppermost internodes. (c) Functional classification of the down‐regulated DEGs in *mir396ef* developing uppermost internode. Numbers in (c) indicate gene numbers. Three independent biological replicates were performed, and error bars indicate standard deviation.

To investigate the mechanisms underlying the shortened internodes in *mir396ef*, we performed RNA‐seq analysis of the developing uppermost internode. A total of 453 DEGs in *mir396ef* (line A1) compared to the wild type (ratio ≥2 or ≤0.5, and FDR <0.05) were observed, including 160 up‐regulated and 293 down‐regulated genes (Data S10). We did not observe any GA biosynthetic, signalling and deactivating genes among these DEGs. GA content measurement in the developing uppermost internodes revealed that *mir396ef* contained a markedly increased level of a bioactive GA precursor GA_20_, whereas other detectable GAs, including GA_7_, GA_19_ and GA_53_, did not show big differences in their levels between *mir396ef* and the wild type (Figure 4a). In the metabolome analyses, which detected 95 compounds with significantly altered levels (FC ≥ 2 or ≤0.5, and VIP ≥ 1; Data S11), MVA level did not show significant changes in the developing uppermost internodes between *mir396ef* and the wild type (FC = 1.23; FC, *mir396ef*/XS134). Based on these results, the internode elongation defect in *mir396ef* cannot be explained by any obvious faults in the GA pathway.

In the transcriptome and real‐time RT‐PCR analyses, we observed that *CYP96B4*, mutation of which severely suppressed internode elongation (Ramamoorthy *et al*., 2011; Tamiru *et al*., 2015; Wang *et al*., 2016; Zhang *et al*., 2014), was significantly down‐regulated in *mir396ef* developing uppermost internode (Figure 4b and Data S10). Loss of *CYP96B4* was reported to result in down‐regulation of the genes involved in carbohydrate and lipid metabolism (Tamiru *et al*., 2015; Wang *et al*., 2016). Enrichment analysis of the DEGs identified in the developing uppermost internodes revealed that many genes involved in carbohydrate and lipid metabolism were significantly down‐regulated in *mir396ef* (Figure 4c and Data S10). These results suggest that *mir396ef* mutations suppress internode elongation via reduced *CYP96B4* expression.

## Discussion

### *MIR396e* and *MIR396f* are valuable gene‐editing targets to breed elite crop varieties

Here, we found that *mir396ef* mutations could enlarge rice grains and panicles, and provided evidence that *mir396ef* mutations could improve productivity. However, compared with the wild type, *mir396ef* displayed an obviously deteriorated shoot architecture. In *mir396ef*, the lengths of leaf blades and sheaths were increased, but the upper three internodes, especially the uppermost internode, were shortened, leading to a defect in panicle exsertion from the flag leaf sheath. Under dry weather condition, this panicle exsertion defect may not affect fertility and seed filling very severely. In Hainan, during the rice‐growing season, humid weather was seldom encountered, and therefore, *mir396ef* showed relatively normal fertility and seed filling. However, in Hangzhou, due to the extremely humid weather, the panicle exsertion defect in *mir396ef* would markedly affect pollination and make the enclosed panicles prone to diseases, thus severely affecting fertility and seed filling. Therefore, this faulty shoot architecture will prevent the application of *mir396ef* mutations in grain yield improvement.

In *mir396ef*, if the shortened internodes are restored to normal or even longer lengths, the plant architecture will be greatly improved. Several genes are known to regulate internode elongation in rice. *ELONGATED UPPERMOST INTERNODE 1* (*EUI1*) encodes a GA‐deactivating enzyme, and *eui1* mutant was morphologically normal until its internodes, especially the uppermost internode, elongated drastically during the heading stage (Luo *et al*., 2006; Zhu *et al*., 2006). HOX12 regulates *EUI1* expression, and knockdown lines of *HOX12* showed a similar morphological phenotype to *eui1* (Gao *et al*., 2016b). Thus, introducing *eui1* or *hox12* mutations into *mir396ef* may overcome the defect in internode elongation and thus improve *mir396ef* shoot architecture to increase productivity.

### *mir396ef* mutations promote leaf elongation through the GA pathway

miR396 negatively regulates leaf growth in *Arabidopsis thaliana* through its targets *GRF* genes (Debernardi *et al*., 2014; Rodriguez *et al*., 2009), and the mechanism underlying this role remains obscure. In this work, we observed longer leaf blades and sheaths in *mir396ef* than in the wild type, indicating that miR396 also negatively regulates leaf growth in rice. Increased GA levels in *mir396ef* leaves suggest that *mir396ef* mutations promote leaf elongation through activating the GA pathway.

MVA is an early precursor of GAs (Ruiz‐Sola *et al*., 2016). Our results suggest that *mir396ef* mutations enhance the GA pathway in leaves through increasing the level of MVA. In plants, MVA is converted to IPP and DMAPP, which are the common precursors of all terpenoids including GAs and ABA (Okada, 2011; Ruiz‐Sola *et al*., 2016). The terpenoid compounds play diverse roles in plants, including attracting insects for pollination, defence against phytopathogenic microbes and enhancing abiotic stress tolerance (Kappers *et al*., 2005; Stoessl *et al*., 1976; Wu *et al*., 2006). Some terpenoids can be used as drugs to combat human diseases (Dewick, 2002). These results imply that *MIR396s* may be involved in plant pollination and stress resistance through controlling terpenoid synthesis, and genetic manipulation of *MIR396s* might boost the production of terpenoids in medicinal plants.

### *mir396ef* mutations modulate shoot architecture through two different pathways

**Figure 5:**
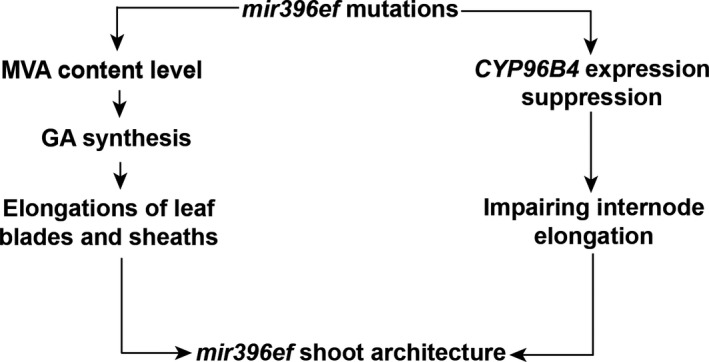
Schematic model showing how *mir396ef* mutations modulate rice shoot architecture. *mir396ef* mutations increase MVA level and thus promote GA synthesis, which further promotes the elongations of leaf blades and sheaths; meanwhile, *mir396ef* mutations decrease *CYP96B4* expression to suppress internode elongation.

Our study suggests that *mir396ef* mutations promote the elongations of leaf blades and sheaths by promoting the GA pathway. However, *mir396ef* impaired the elongation of the upper three internodes in the stem, especially the uppermost internode. We did not find any obvious defects in the GA pathway that may explain the suppressed internode elongation in *mir396ef* mutant plants. Mutation of *CYP96B4* was reported to suppress internode elongation (Ramamoorthy *et al*., 2011; Tamiru *et al*., 2015; Wang *et al*., 2016; Zhang *et al*., 2014). In *mir396ef* developing uppermost internode, the markedly decreased expression of *CYP96B4* suggests that *mir396ef* mutations impair internode elongation through suppressing *CYP96B4* expression. Therefore, our results suggest that *mir396ef* mutations modulate shoot architecture through two different pathways (Figure 5). The reasons for the opposite effects of *mir396ef* mutations on the internodes and leaves, as well as how miR396 regulates *CYP96B4* expression, remain to be revealed in the future.

### *GRF* genes are involved in forming the phenotypic traits caused by *mir396ef* mutations

Previous reports identified a major grain size controlling QTL *GS2*/*GL2* (*GRF4*), one miR396 target gene (Che *et al*., 2016; Duan *et al*., 2016; Gao *et al*., 2016a; Hu *et al*., 2015; Li *et al*., 2016). The phenotype caused by *mir396ef* mutations shows some similarities with that induced by the large‐grain *GS2*/*GL2* allele (*GRF4* gain‐of‐function allele), such as lengthened leaves, enlarged grains and increased panicle size. These similarities are reasonable partly because *GRF4* expression level is increased in *mir396ef* compared with the wild type (Figure S5a, b). However, we observed shortened upper three internodes in *mir396ef*, but similar phenotype was not reported in plants with the *GRF4* gain‐of‐function allele. In addition, the up‐regulation of *GRF4* in *mir396ef* leaves and uppermost internodes is not very strong (Figure S5a, b). These results strongly suggest that other miR396 target genes are also involved in the phenotypic traits we observed in *mir396ef*. Our transcriptome analyses in leaves and developing uppermost internodes revealed that several miR396‐targeting *GRF* genes, especially *GRF3*, were up‐regulated by *mir396ef* mutations (Figure S5a, b). Among the miR396‐targeting *GRFs*,* GRF3* showed the highest expression level in both wild‐type and *mir396ef* leaves and uppermost internodes, and was up‐regulated by *mir396ef* mutations more intensely than most other *GRF* genes (Figure S5a, b), suggesting that *GRF3* is intensely involved in the changed elongations of leaves and internodes in *mir396ef*. How *GRF3* regulates the elongations of leaves and internodes remains to be revealed in the future.

## Experimental procedures

### Vector construction and plant cultivation

A multiplex gene‐editing vector was constructed to edit *MIR396a*,* MIR396b*,* MIR396c*,* MIR396e* and *MIR396f*, and in this vector, four sgRNA expression cassettes were arranged in tandem (Figure 1a). To mutate *MIR396*s individually, seven single sgRNA‐expressing vectors were also constructed, among which one vector targets *MIR396e* and *MIR396f*, and the others are single *MIR396* gene‐targeting vectors. All these vectors are specific for the *MIR396* gene sequences. The vectors were transformed into XS134 through *Agrobacterium*‐mediated transformation. The transgenic plants were grown in paddy fields under natural conditions. Because the sgRNAs designed for certain *MIR396* genes may induce off‐target mutations in other *MIR396* genes, we sequenced both the target sites and potential off‐target sites in *MIR396* genes to accurately identify the genotype. The seeds from the transgenic plants were sowed in the Lingshui County of Hainan Province (China) in late December and in Hangzhou (China) in early June.

### Small RNA northern blot analysis

Small RNA northern blots were performed as previously described (Pall and Hamilton, 2008) with minor modifications. In brief, twenty μg total RNA was separated by running a 15% SDS‐PAGE with 7 M urea and then transferred to a Hybond NX membrane (GE, Amersham). After that, a 1‐ethyl‐3‐(3‐dimethylaminopropyl) carbodiimide (EDC)‐mediated chemical cross‐linking was carried out as previously described (Pall and Hamilton, 2008). Antisense complementary oligonucleotides for miRNAs and U6 probes were end‐labelled ([γ‐^32^P] ATP) by T4 polynucleotide kinase (*New England Biolabs*). The probe sequences are listed in Table S2.

### Epidermal cell observation and sectioning analyses

For histological sectioning and epidermal cell observation, the materials were first fixed in FAA's solution (50% ethanol, 5% glacial acetic acid and 5% formaldehyde). For epidermal cell observation, fresh or fixed leaf blades and sheaths were soaked in boiled water for 10 min, and then transferred to 95% ethanol and boiled for about 1 h until being completely faded. Then, the materials were soaked in 85% lactic acid of 96 °C for 8 min. After cooling to room temperature, the epidermal layers were observed under a microscope. For sectioning analyses, after being dehydrated in a graded ethanol series (70%, 80%, 90% and 100%), the fixed samples were embedded in paraffin. The embedded samples were then sliced into 10–15 μm sections for observation. The transverse sections and epidermal cells were imaged with a Leica DM2500 microscope (Leica Microsystems).

### Plot field tests

Plants of the wild type and *mir396ef* were grown in Hangzhou and Hainan paddy fields under natural conditions. The area per plot was 90 × 60 cm, and 24 plants were cultivated in each plot with planting density of 15 × 15 cm.

### Real‐time RT‐PCR

The total RNA was extracted using the TRIzol^™^ Reagent (Invitrogen, Cat. no. 15596018). Reverse transcription was performed using the SuperScript^®^ III Reverse Transcriptase (Invitrogen, Cat. no. 18080‐044). Real‐time PCR analyses were performed using the Bio‐Rad CFX96 real‐time PCR instrument and EvaGreen (Biotium, Cat. no. 31000). The PCR was conducted with gene‐specific primers for the target genes (Table S3), and *UBIQUITIN* was used as the reference gene in the real‐time RT‐PCR.

### Transcriptome analyses

The materials were sampled with three biological repeats for RNA‐sequencing (RNA‐seq) analyses. RNAs were extracted with RNAprep pure Plant kit (TIANGEN, Cat. no. DP432), and then, libraries were constructed using TruSeq Stranded mRNA (Illumina, San Diego, CA) in accordance with the manufacturer's instruction. Qualities of RNA‐seq libraries were assessed by using a Fragment Analyzer (Advanced Analytical, IA), and resulting libraries were sequenced using Illumina Hiseq X ten. The raw reads were filtered by removing reads containing adapter and low‐quality reads for subsequent analyses. Clean reads were aligned to the rice reference genome (TIGR release 7) using Hisat2 with default parameters, and resultant files were input to the Cufflinks software for comparative assembly of transcripts and generation of fragments per kilobase of exon per million reads mapped (FPKM). Subsequently, gene expression analyses between the wild type and *mir396ef* were executed using the cufflinks‐cuffdiff analysis module.

### Phytohormone measurement

Plant materials were ground into powder in liquid nitrogen and extracted with methanol/water (8/2) at 4°C. The extract was centrifuged at 12 000 *g* under 4°C for 15 min. The supernatant was collected and evaporated to dryness under nitrogen gas stream, and then reconstituted in methanol/water (3/7). The solution was centrifuged, and the supernatant was collected for LC‐MS analysis. The LC‐MS analysis was conducted with the API6500 Q TRAP LC/MS/MS system, equipped with an ESI Turbo Ion‐Spray interface, operating in a positive ion mode and controlled by Analyst 1.6 software (AB Sciex).

### Metabolome analyses

The metabolome analyses were conducted as previously described (Wang *et al*., 2017). The freeze‐dried materials were crushed to powder, and then, 100 mg powders were weighted and extracted overnight at 4°C with 1 mL 70% aqueous methanol. Following centrifugation at 10 000 *g* for 10 min, the extracts were filtrated (SCAA‐104, 0.22 μm pore size; ANPEL) before LC‐MS analysis.

The sample extracts were analysed using an LC‐ESI‐MS/MS system (HPLC, Shim‐pack UFLC SHIMADZU CBM30A system; MS, Applied Biosystems 4500 Q TRAP). The analytical conditions were as follows: HPLC: column, Waters ACQUITY UPLC HSS T3 C18 (1.8 μm, 2.1 × 100 mm); solvent system, water (0.04% acetic acid): acetonitrile (0.04% acetic acid); gradient program, 100:0 V/V at 0 min, 5:95 V/V at 11.0 min, 5:95 V/V at 12.0 min, 95:5 V/V at 12.1 min, 95:5 V/V at 15.0 min; flow rate, 0.4 mL/min; temperature, 40 °C; and injection volume, 5 μL. The effluent was alternatively connected to an ESI‐triple quadrupole‐linear ion trap Q TRAP‐MS.

LIT and triple quadrupole (QQQ) scans were acquired on a triple quadrupole‐linear ion trap mass spectrometer (Q TRAP), API 4500 Q TRAP LC/MS/MS system, equipped with an ESI Turbo Ion‐Spray interface, operating in a positive ion mode and controlled by Analyst 1.6 software (AB Sciex). The ESI source operation parameters were as follows: ion source, turbo spray; source temperature, 550°C; ion spray voltage (IS), 5500 V; ion source gas I (GSI), gas II(GSII), curtain gas (CUR) were set at 55, 60 and 25 psi, respectively; the collision gas (CAD) was high. Instrument tuning and mass calibration were performed with 10 and 100 μmol/L polypropylene glycol solutions in QQQ and LIT modes, respectively. QQQ scans were acquired as MRM experiments with collision gas (nitrogen) set to 5 psi. DP and CE for individual MRM transitions were done with further DP and CE optimization. A specific set of MRM transitions was monitored for each period according to the metabolites eluted within this period.

## Accession numbers

The sequence data of the *MIR396* genes can be found in the miRbase database (http://www.mirbase.org/) under the following accession numbers: *MIR396a*, MI0001046; *MIR396b*, MI0001047; *MIR396c*, MI0001048; *MIR396d*, MI0013049; *MIR396e*, MI0001703; *MIR396f*, MI0010563; *MIR396g*, MI0013047; and *MIR396h*, MI0013048. The sequence data of the other genes can be found in the MSU database (http://rice.plantbiology.msu.edu/) under the following gene locus identifiers: *GRF1*, LOC_Os02g53690; *GRF2*, LOC_Os06g10310; *GRF3*, LOC_Os04g51190; *GS2*/*GL2*/*GRF4*, LOC_Os02g47280; *GRF5*, LOC_Os06g02560; *GRF6*, LOC_Os03g51970; *GRF7*, LOC_Os12g29980; *GRF8*, LOC_Os11g35030; *GRF9*, LOC_Os03g47140; *GRF10*, LOC_Os02g45570; *GRF12*, LOC_Os04g48510; *GA20ox1*, LOC_Os03g63970; *GA20ox2*, LOC_Os01g66100; *GA20ox3*, LOC_Os07g07420; *GA20ox4*, LOC_Os05g34854; *GA3ox1*, LOC_Os05g08540; *GA3ox2*, LOC_Os01g08220; *CPS1*, LOC_Os02g17780; *KS1*, LOC_Os04g52230; *KAO*, LOC_Os06g02019; *KO2*, LOC_Os06g37364; *GID1L2*, LOC_Os03g57640; *GID1L3*, LOC_Os07g34370; *GA2ox1*, LOC_Os05g06670; *GA2ox3*, LOC_Os01g55240; *GA2ox6*, LOC_Os04g44150; *GA2ox7*, LOC_Os01g11150; *GA2ox8*, LOC_Os05g48700; *GA2ox9*, LOC_Os02g41954; *TPS3*, LOC_Os08g04500; *TPS20*, LOC_Os04g27340; *TPS24*, LOC_Os04g27790; *CYP96B4*, LOC_Os03g04680.

## Conflict of interest

The authors declare no conflict of interest.

## Author contributions

C.M. and D.W. conceived and designed the research. C.M. constructed the plant materials, performed the phytohormone measurements and conducted the phenotypic, transcriptome and metabolome analyses. D.W. and R.H. conducted the northern blotting and real‐time RT‐PCR analyses. C.M. and D.W. did the other assays together. All the authors analysed the data together. C.M. and D.W. wrote the manuscript together. C.M., D.W. and J.‐K.Z. oversaw the entire study.

## Supporting information


**Figure S1.** *MIR396‐GRF* gene structures and target sites of *MIR396* gene editing.

**Figure S2.** Comparison of wild‐type and *mir396* seedlings.

**Figure S3.** Detection of miR396e and miR396f in seedling shoots by Northern blotting analyses.

**Figure S4.** *mir396ef* mutations increase the cell lengths of leaf blades and sheaths but decrease the cell length of uppermost internode.

**Figure S5.** Relative expression analyses of miR396 target genes in leaves and developing uppermost internodes.

**Figure S6.** Seed size and fertility analyses of the wild type, *mir396ef* and *mir396abcef* in Hangzhou.

**Figure S7.** *mir396ef* mutations enlarged the cells of spikelet hulls.

**Figure S8.** Real‐time RT‐PCR analyses of *MIR396e* and *MIR396f* expressions.

**Figure S9.** Grain yield investigation of the wild type and *mir396ef* in Hangzhou.

**Figure S10.** Expression profiles of GA biosynthetic, signaling, deactivating and response genes in the leaves of 50‐day‐old wild‐type and *mir396ef* plants.

**Figure S11.** Terpenoid biosynthetic pathway (Ruiz‐Sola *et al*., 2016).

**Figure S12.** Endogenous ABA and CK levels in the leaves of 50‐day‐old wild‐type and *mir396ef* plants.

**Table S1.** The up‐regulated DEG (ratio ≥ 2, and FDR < 0.05) *TPSs* in the leaves of 50‐day‐old *mir396ef* plants.
**Table S2.** Probes used in the Northern blot assays.
**Table S3.** Primers for real‐time RT‐PCR.

**Data S1.** The list of rice *mir396* mutants.

**Data S2.** Gene expression profiles in wild‐type and *mir396ef* (line A1) leaves.

**Data S3.** Gene expression profiles in wild‐type and *mir396ef* (line A6) developing uppermost internodes.

**Data S4.** Expression profiles of the DEGs identified in wild‐type and *mir396ef* (line A1) leaves.

**Data S5.** Expression profiles of the GA‐related genes in wild‐type and *mir396ef* (line A1) leaves.

**Data S6.** Expression profiles of the DEGs identified in wild‐type and *mir396ef* (line A6) leaves.

**Data S7.** Expression profiles of the GA‐related genes in wild‐type and *mir396ef* (line A6) leaves.

**Data S8.** The metabolites detectable in the metabolome analyses.

**Data S9.** The metabolites with significantly different levels between wild‐type and *mir396ef* leaves.

**Data S10.** Expression profiles of the DEGs identified in wild‐type and *mir396ef* developing uppermost internodes.

**Data S11.** The metabolites with significantly different levels between wild‐type and *mir396ef* developing uppermost internodes.
