# Supplementary figures and images for: Mutations in MIR396e and MIR396f increase grain size and modulate shoot architecture in rice
Source: Plant Biotechnol J. 2019 Aug 16;18(2):491–501. doi: 10.1111/pbi.13214 (PMC6953237; doi:10.1111/pbi.13214)

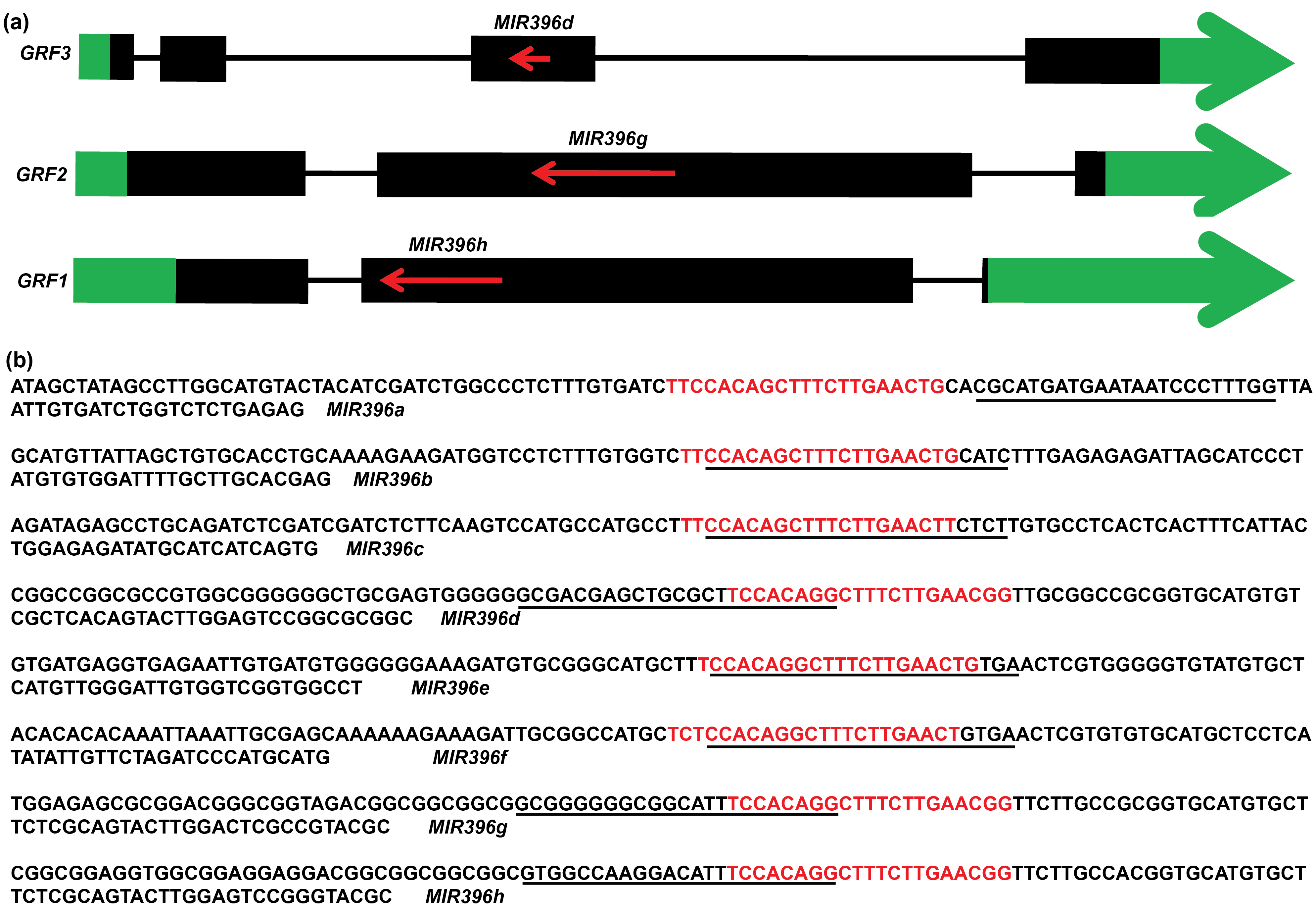

Supplement: Supplementary file 1 — Figure S1. MIR396‐GRF gene structures and target sites of MIR396 gene editing. [file PBI-18-491-s021.png]

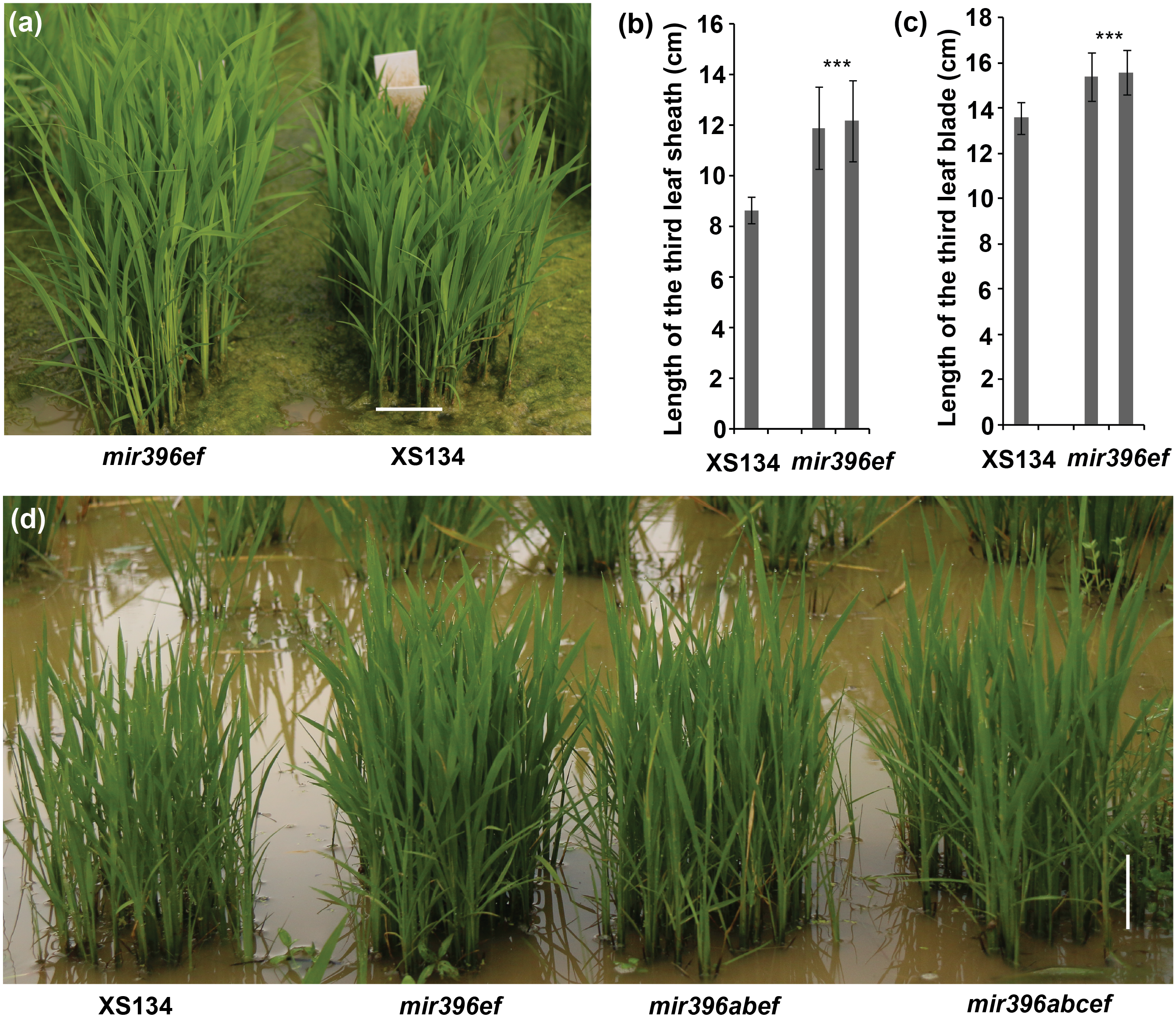

Supplement: Supplementary file 2 — Figure S2. Comparison of wild‐type and mir396 seedlings. [file PBI-18-491-s020.png]

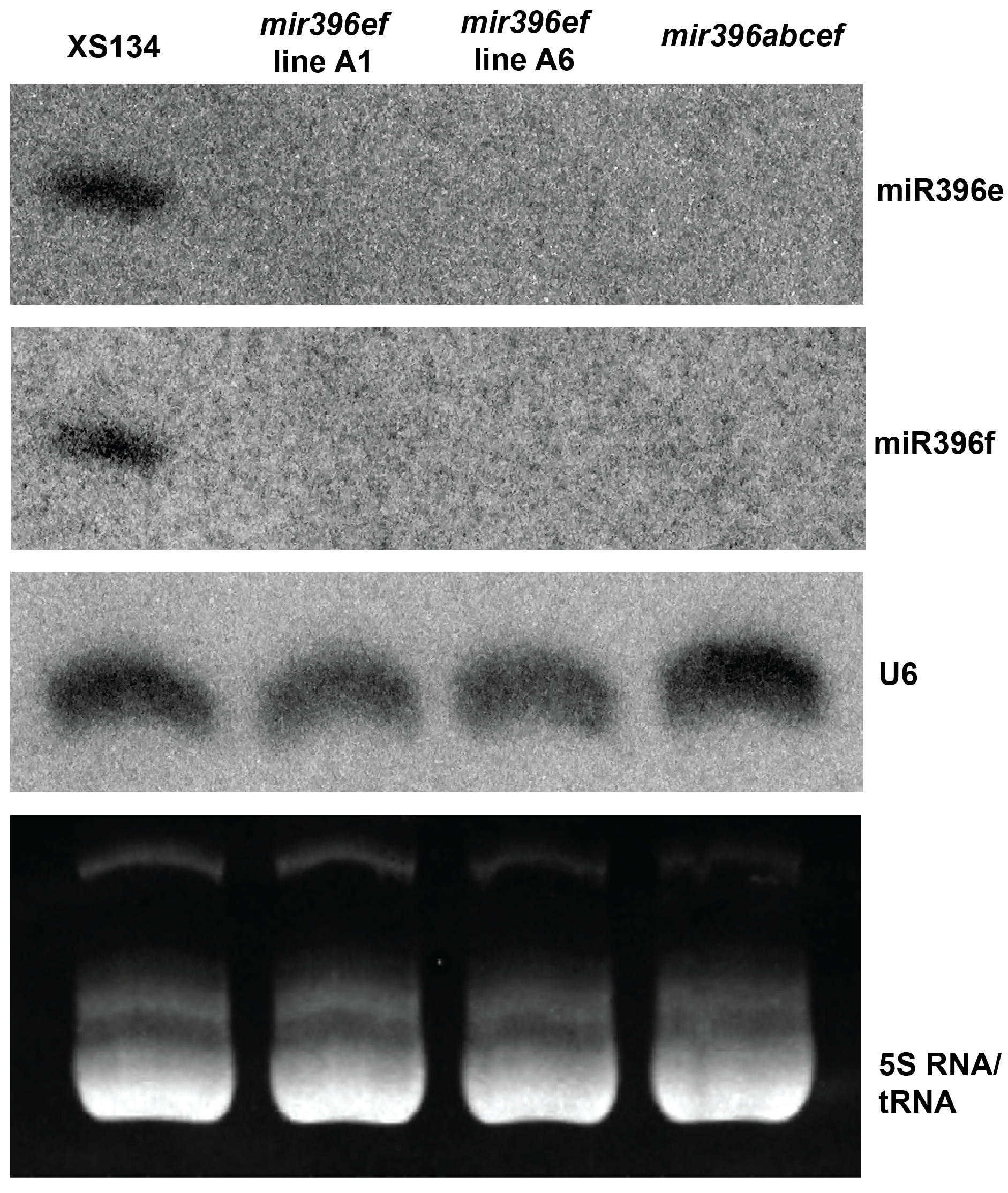

Supplement: Supplementary file 3 — Figure S3. Detection of miR396e and miR396f in seedling shoots by Northern blotting analyses. [file PBI-18-491-s019.png]

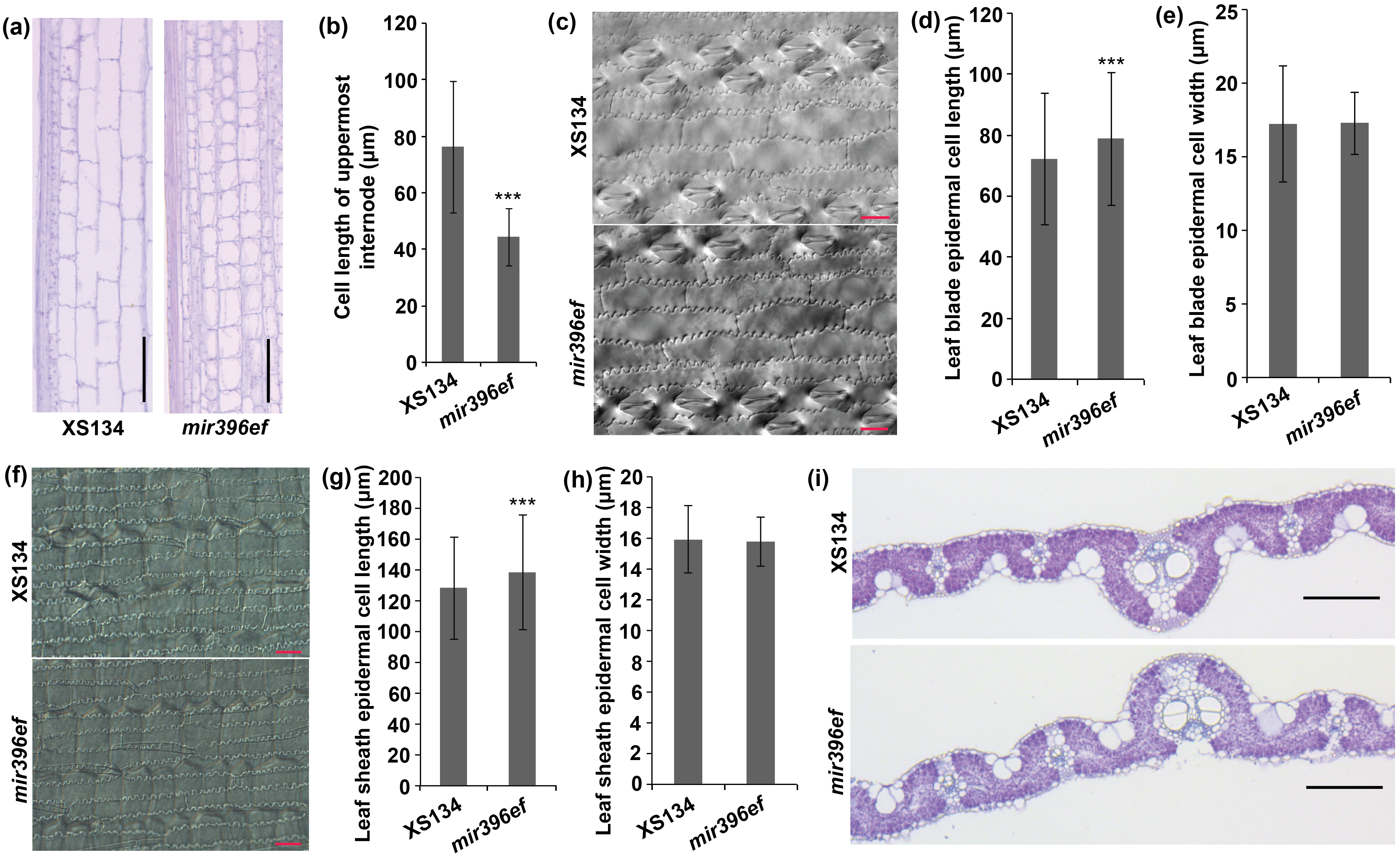

Supplement: Supplementary file 4 — Figure S4. mir396ef mutations increase the cell lengths of leaf blades and sheaths but decrease the cell length of uppermost internode. [file PBI-18-491-s023.png]

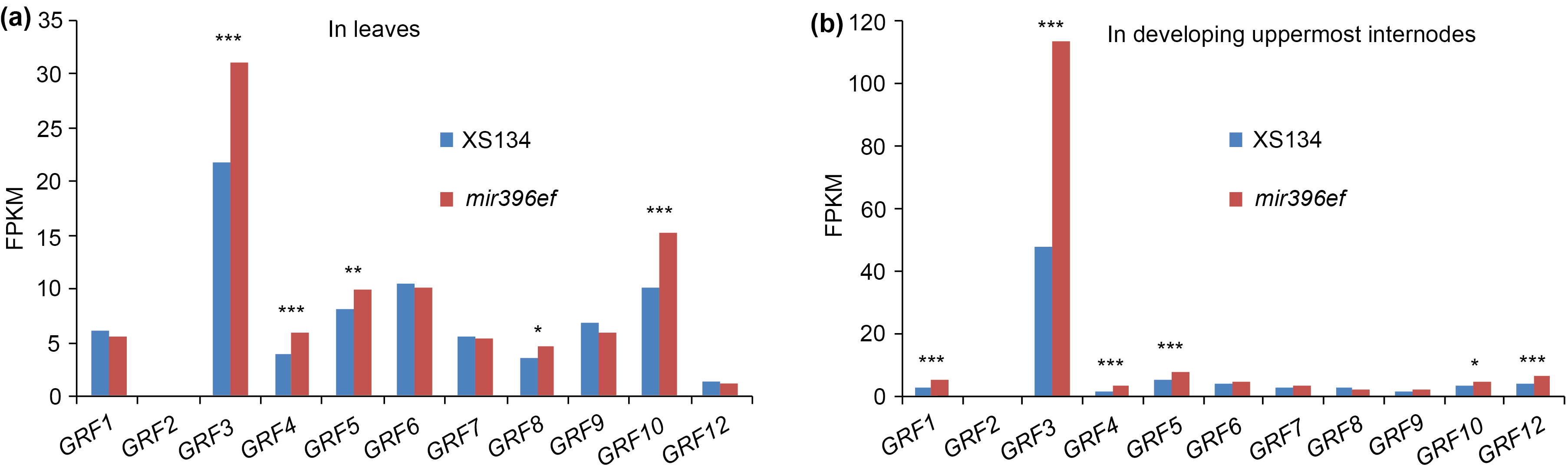

Supplement: Supplementary file 5 — Figure S5. Relative expression analyses of miR396 target genes in leaves and developing uppermost internodes. [file PBI-18-491-s024.png]

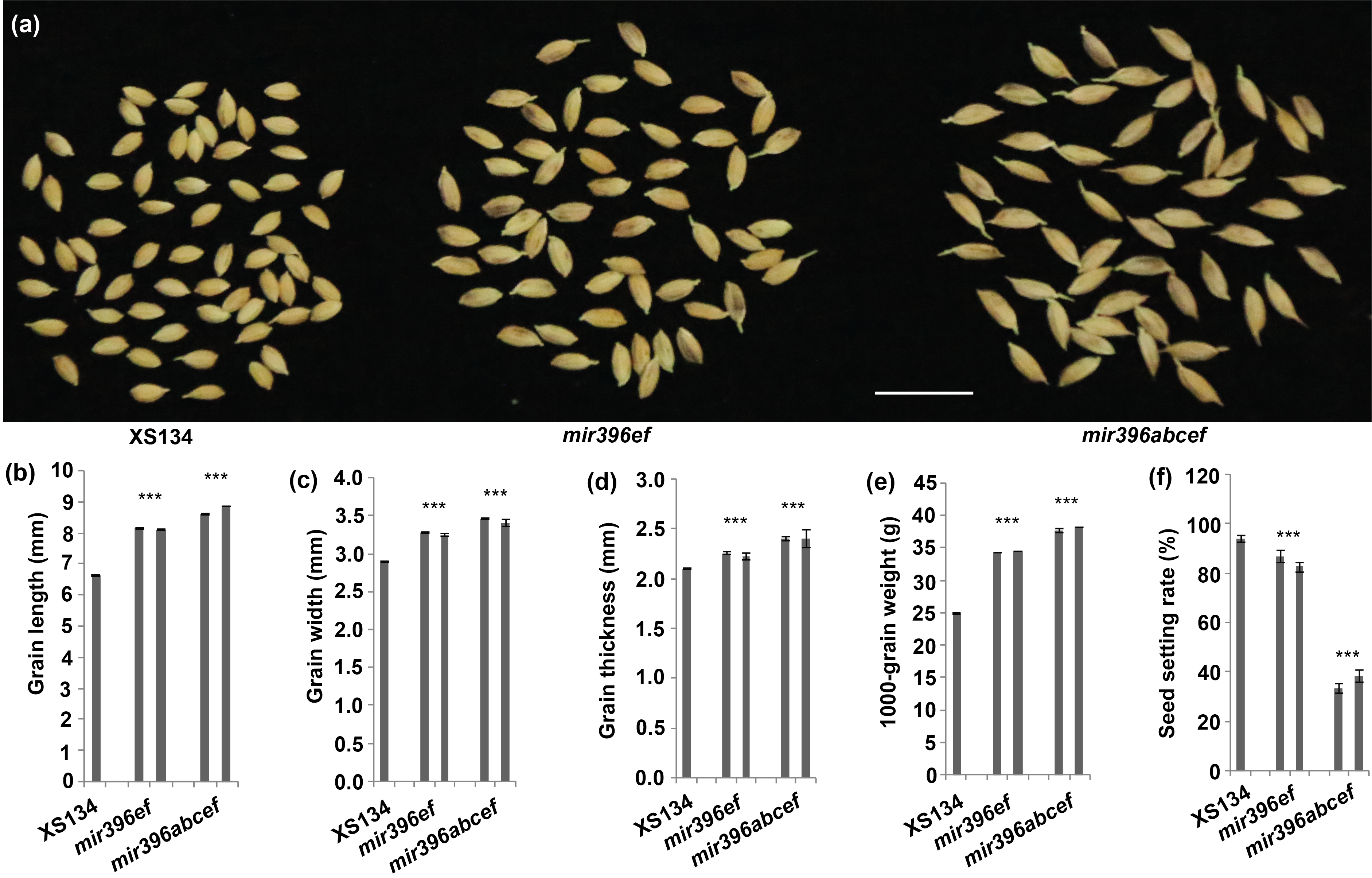

Supplement: Supplementary file 6 — Figure S6. Seed size and fertility analyses of the wild type, mir396ef and mir396abcef in Hangzhou. [file PBI-18-491-s001.png]

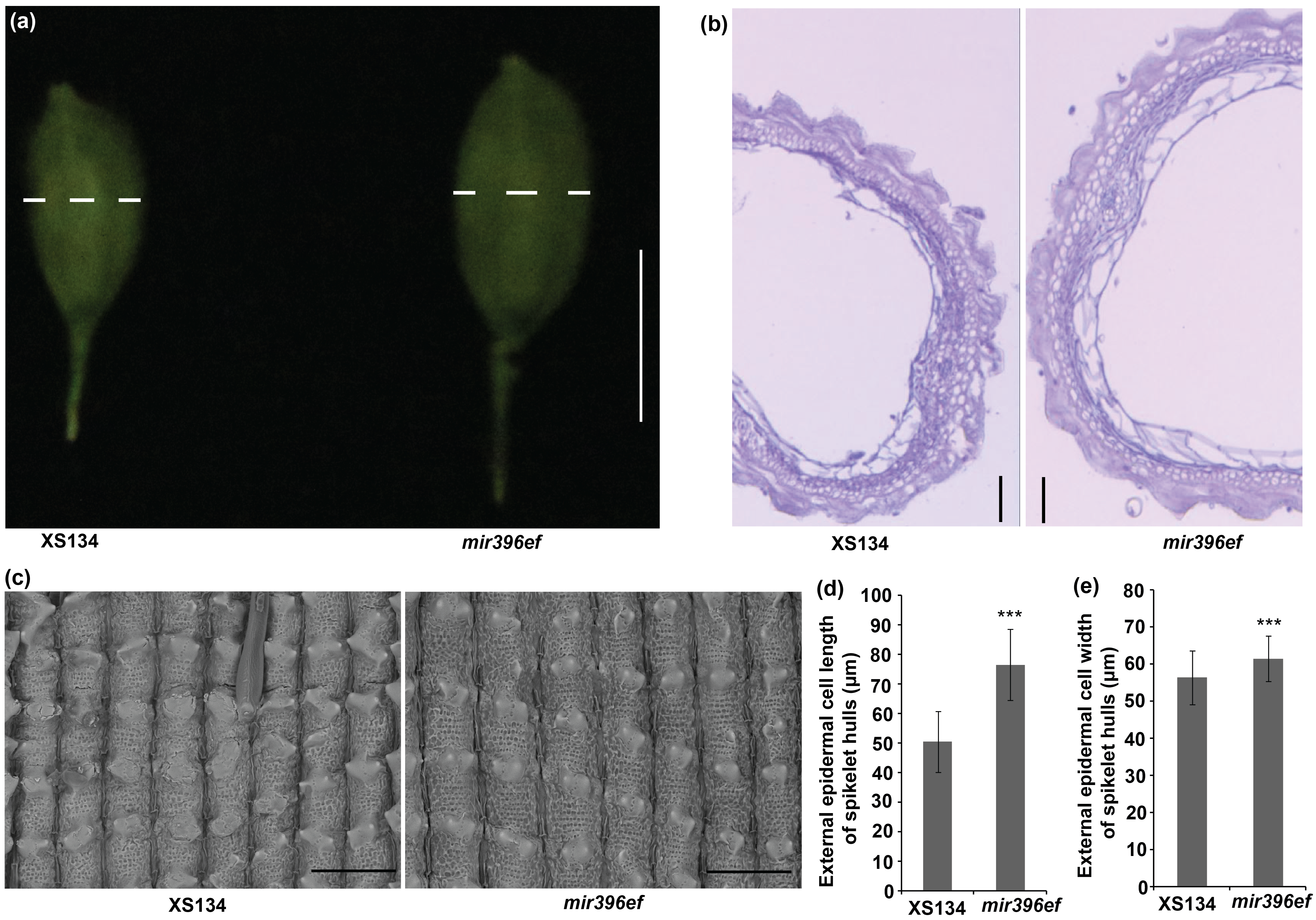

Supplement: Supplementary file 7 — Figure S7. mir396ef mutations enlarged the cells of spikelet hulls. [file PBI-18-491-s002.png]

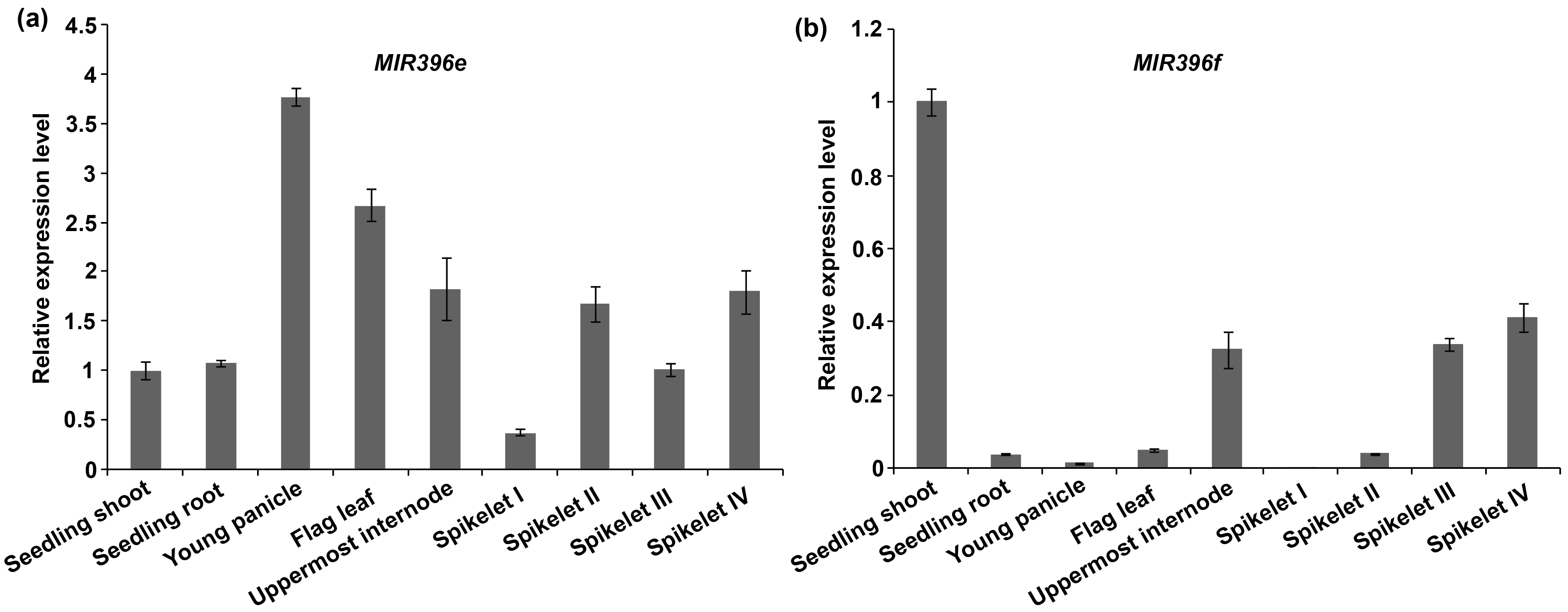

Supplement: Supplementary file 8 — Figure S8. Real‐time RT‐PCR analyses of MIR396e and MIR396f expressions. [file PBI-18-491-s003.png]

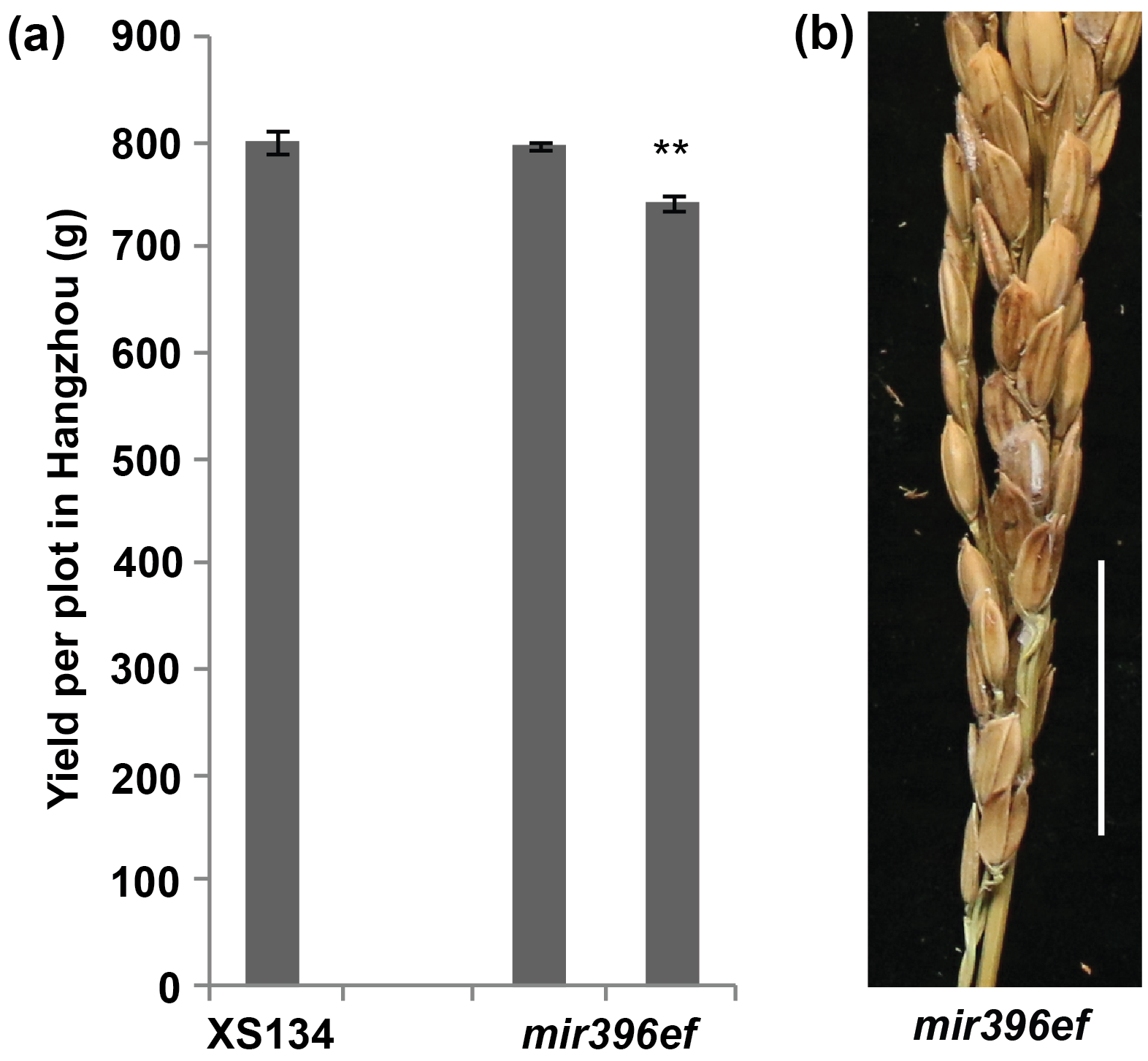

Supplement: Supplementary file 9 — Figure S9. Grain yield investigation of the wild type and mir396ef in Hangzhou. [file PBI-18-491-s004.png]

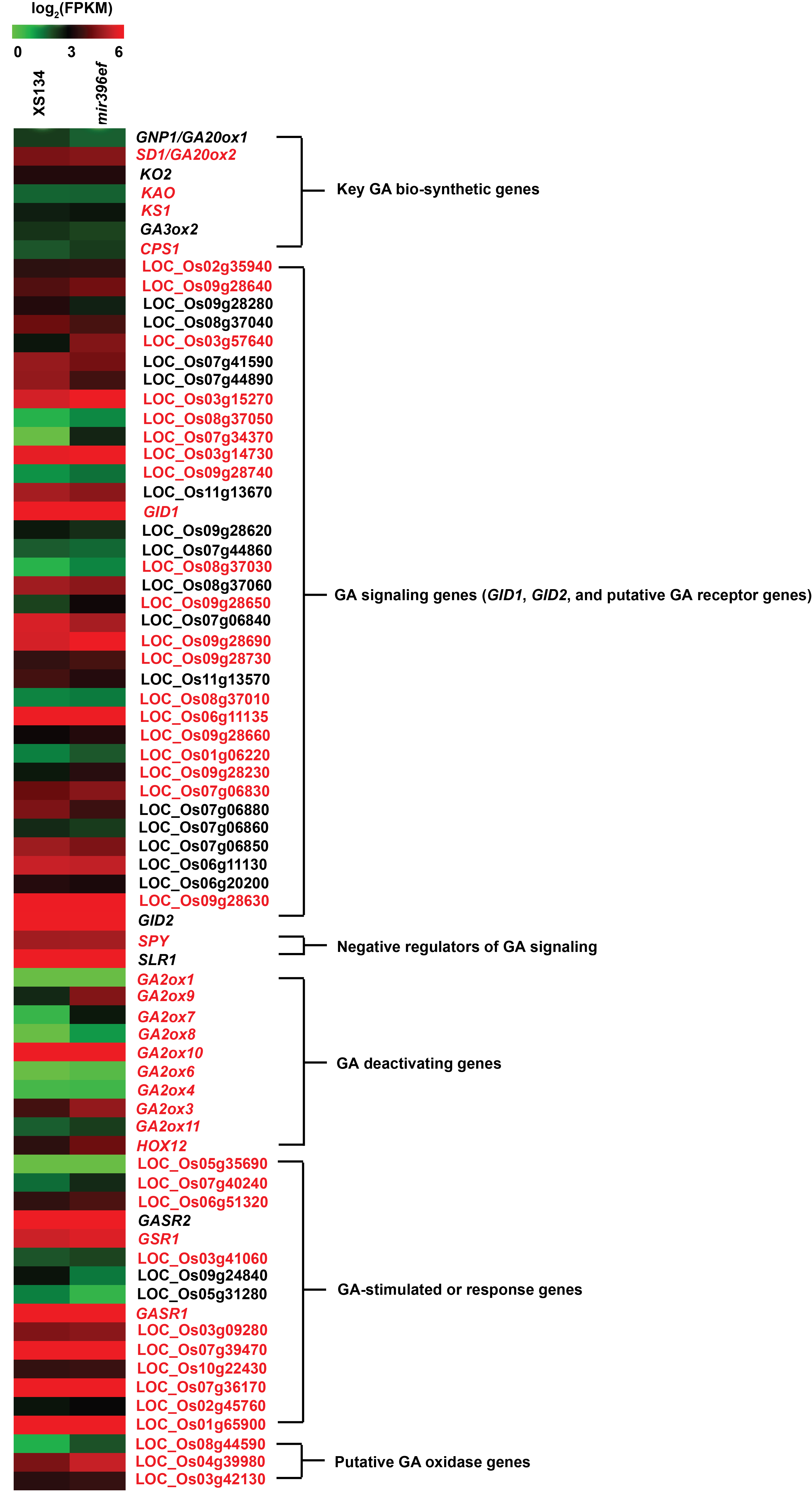

Supplement: Supplementary file 10 — Figure S10. Expression profiles of GA biosynthetic, signaling, deactivating and response genes in the leaves of 50‐day‐old wild‐type and mir396ef plants. [file PBI-18-491-s005.png]

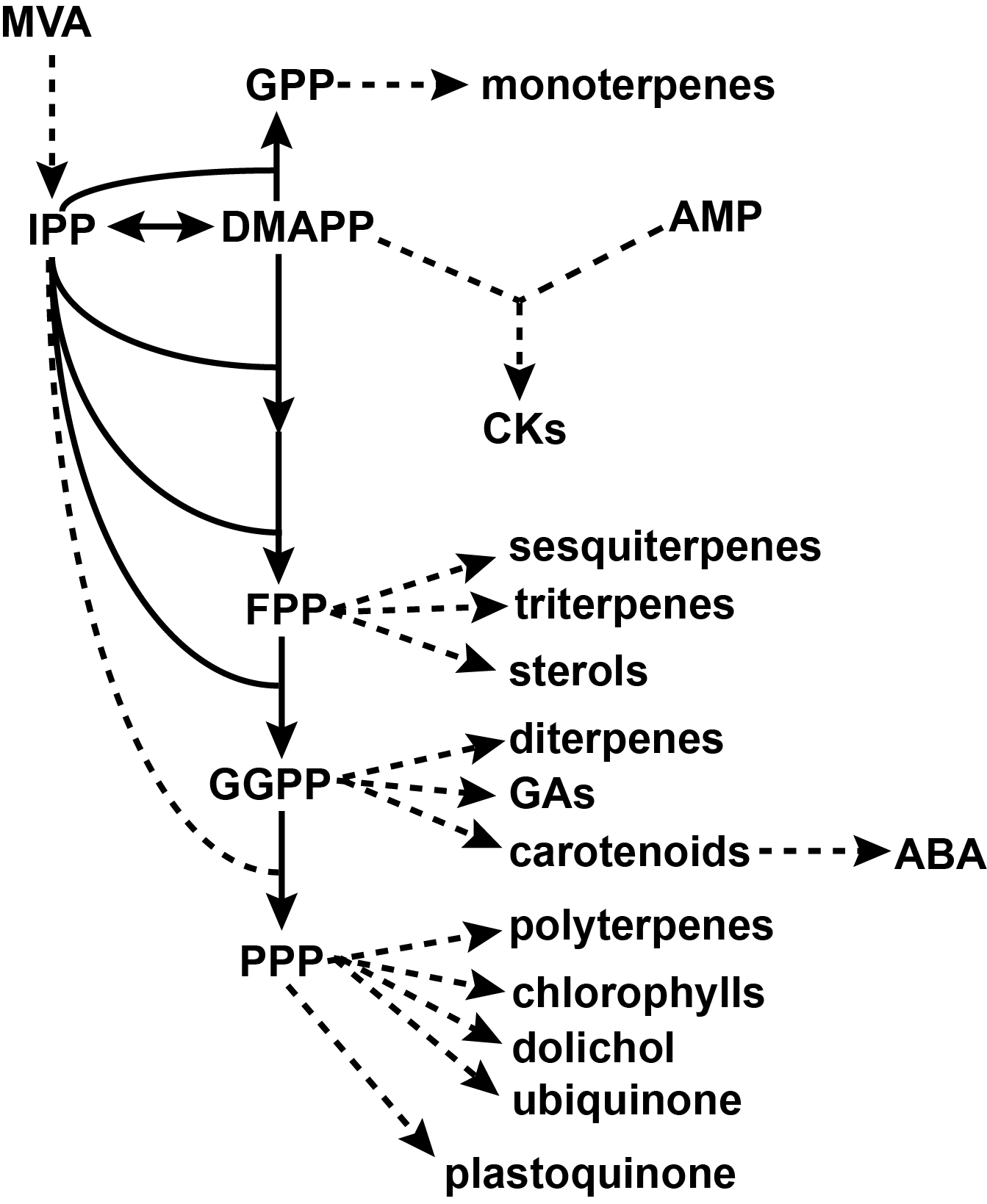

Supplement: Supplementary file 11 — Figure S11. Terpenoid biosynthetic pathway (Ruiz‐Sola et al., 2016). [file PBI-18-491-s006.png]

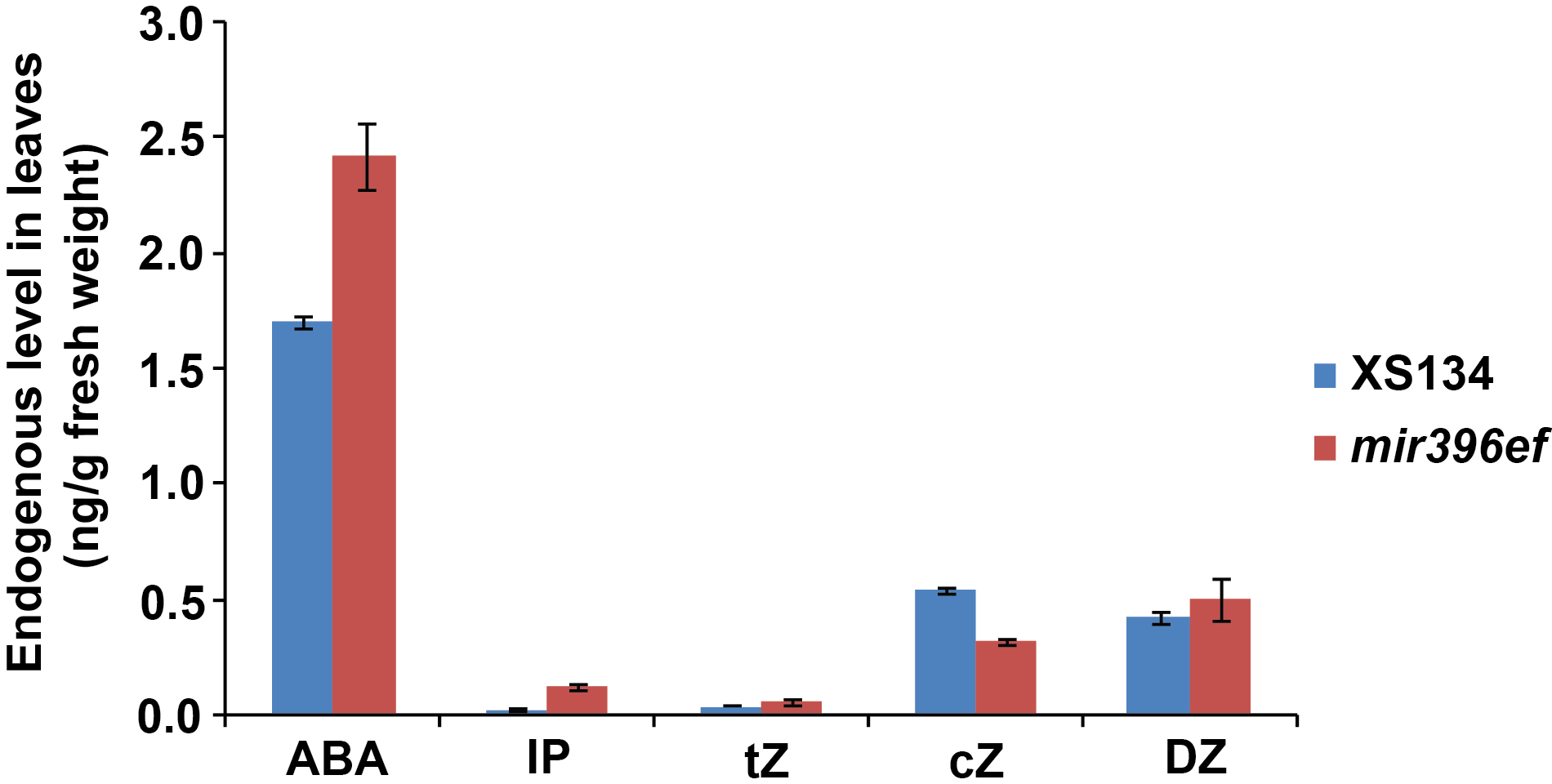

Supplement: Supplementary file 12 — Figure S12. Endogenous ABA and CK levels in the leaves of 50‐day‐old wild‐type and mir396ef plants. [file PBI-18-491-s007.png]
