# Supplementary material for: Mutations in MIR396e and MIR396f increase grain size and modulate shoot architecture in rice
Source: Plant Biotechnol J. 2019 Aug 16;18(2):491–501. doi: 10.1111/pbi.13214 (PMC6953237; doi:10.1111/pbi.13214)
Supplement: Supplementary file 13 — Table S1. The up‐regulated DEG (ratio ≥ 2, and FDR < 0.05) TPSs in the leaves of 50‐day‐old mir396ef plants. Table S2. Probes used in the Northern blot assays. Table S3. Primers for real‐time RT‐PCR. [file PBI-18-491-s008.docx]

**Table S1.** The up-regulated DEG (ratio ≥ 2, and FDR ＜ 0.05) *TPSs* in the leaves of 50-day-old *mir396ef* plants

| Lines | Genes | Annotations |
| --- | --- | --- |
| A1 | *TPS20* (LOC_Os04g27340) | Terpene synthase responsible for producing monoterpene volatiles in response to abiotic stresses |
|  | LOC_Os02g02930 | Terpene synthase, putative, expressed |
|  | LOC_Os10g34790 | Terpene synthase, putative, expressed |
|  | LOC_Os04g27430 | Terpene synthase, putative, expressed |
|  | LOC_Os08g07100 | Terpene synthase, putative, expressed |
|  | LOC_Os11g28530 | Terpene synthase, putative, expressed |
|  | LOC_Os08g07080 | Terpene synthase, putative, expressed |
|  | LOC_Os02g36264 | Terpene synthase, putative, expressed |
|  | LOC_Os03g22620 | Terpene synthase, putative, expressed |
|  | LOC_Os04g52210 | Terpene synthase, putative, expressed |
|  | LOC_Os02g26014 | Terpene synthase, putative, expressed |
|  | *TPS3* (LOC_Os08g04500) | Terpene synthase responsible for producing jasmonate-induced sesquiterpenes |
|  | *TPS24* (LOC_Os04g27790) | *TPS24* encodes a jasmonate-responsive monoterpene synthase that produces an antibacterial γ-terpinene against rice pathogen |
|  | LOC_Os03g22634 | Terpene synthase, putative, expressed |
| A6 | *TPS20* (LOC_Os04g27340) | Terpene synthase responsible for producing monoterpene volatiles in response to abiotic stresses |
|  | LOC_Os02g02930 | Terpene synthase, putative, expressed |
|  | LOC_Os10g34790 | Terpene synthase, putative, expressed |
|  | LOC_Os04g27430 | Terpene synthase, putative, expressed |
|  | LOC_Os08g07100 | Terpene synthase, putative, expressed |
|  | LOC_Os11g28530 | Terpene synthase, putative, expressed |
|  | LOC_Os08g07080 | Terpene synthase, putative, expressed |
|  | LOC_Os02g36220 | Terpene synthase, putative, expressed |
|  | LOC_Os04g01810 | Terpene synthase, putative, expressed |
|  | LOC_Os04g27670 | Terpene synthase, putative, expressed |
|  | LOC_Os04g27190 | Terpene synthase, putative, expressed |

The data were taken from the transcriptome analyses of the wild type and *mir396ef*.

**Table S2.** Probes used in the Northern blot assays

| Probes | Sequences (5’ to 3’) |
| --- | --- |
| anti-miR396e | CAGTTCAAGAAAGCCTGTGGA |
| anti-miR396f | AGTTCAAGAAAGCCTGTGGAGA |
| anti-U6 | TGTATCGTTCCAATTTTATCGGATGT |

**Table S3.** Primers for real-time RT-PCR

| Genes | Primers | Sequences (5’ to 3’) |
| --- | --- | --- |
| *UBIQUITIN* (LOC_Os03g13170) | Ubi-F | GTACAACCAGGACAAGATGATCTG |
|  | Ubi-R | AGATAACAACGGAAGCATAAAAGTC |
| *MIR396e* | MIR396e-F | ACTGTGAACTCGTGGGGGTGT |
|  | MIR396e-R | CATCAACAGGTAGAGACGACCAA |
| *MIR396f* | MIR396f-F | CGTGTGTGCATGCTCCTCATA |
|  | MIR396f-R | ACAAGATGGGGATCCAGATGC |
| *GA20ox1* | GA20ox1-F | TACTCGCGCTACTGCCACGAG |
|  | GA20ox1-R | GCACGCCGGGTAGTAGTTGAG |
| *GA20ox2* | GA20ox2-F | ATTTTGGACCCTACCGCTGT |
|  | GA20ox2-R | TAGAGAGAAGCCCAACCCAAC |
| *GA20ox3* | GA20ox3-F | TTCACGCAGAGGCACTACCG |
|  | GA20ox3-R | CCTGATCTTCTCATCCCATCTGTC |
| *GA20ox4* | GA20ox4-F | CGCCGATTACTTCTCCACCCTA |
|  | GA20ox4-R | CTCCCCTAGCACCGCCATTATC |
| *GA3ox1* | GA3ox1-F | GGAGAGCAAGGCCGTGTATCA |
|  | GA3ox1-R | CCTTGTCCTCTTCCTTCGCTACTC |
| *GA3ox2* | GA3ox2-F | CTCCTTCTTCTCCAAGCTCATGT |
|  | GA3ox2-R | CAAGAACAACCTCAGCAACTCGT |
| *GA20ox7* | GA20ox7-F | ATATTGAGCAATGGACGGTACAGG |
|  | GA20ox7-R | GAAGGTGGCGAGGTCGGTGT |
| *CPS1* | CPS1-F | TTGCTTCCTCTATTTGCGATTC |
|  | CPS1-R | TTCACAATGCTCAAGAAGGTCTG |
| *KS1* | KS1-F | TGCTCCCGACTCTGTATTTCGTAG |
|  | KS1-R | GAACCACCACTGTGATGAACAAGC |
| *KAO* | KAO-F | TTTGGACTGGGAGCAAGACTGT |
|  | KAO-R | TAGTGTTCATCGGAAACTTTGGTG |
| *KO2* | KO2-F | TGGTTGCTACCAGCGACTATTG |
|  | KO2-R | GGGTCATCTTTCACCAGTTTATGAA |
| *GID1L2* | GID1L2-F | ACTGAGCTGCCTGAGTCCACTTC |
|  | GID1L2-R | CTGGAGGTTAGGGCCGCAGT |
| *GID1L3* | GID1L3-F | GCAGACAAGCAGGACGCTCA |
|  | GID1L3-R | ATCACGAAACGCAGCAAGGTAC |
| *GA2ox1* | GA2ox1-F | CTCTGACAAATGGGAGGCTGAT |
|  | GA2ox1-R | GCTGCTGGCTGTGATTGTCTC |
| *GA2ox3* | GA2ox3-F | ACTCGTTGCAGGTTCTGACCAA |
|  | GA2ox3-R | AATGGTGCAATCCTCTGTGCTAA |
| *GA2ox6* | GA2ox6-F | GGAGCAACAACAGGTACAAGAGCG |
|  | GA2ox6-R | AAGCGAGTCGTAGGACGGGCA |
| *GA2ox7* | GA2ox7-F | ATCTCCGTGCTCCGCTCCAA |
|  | GA2ox7-R | TTCACCACCACCCTGTGCTTCA |
| *GA2ox8* | GA2ox8-F | TCATCTCCGTGCTCAGGTCCAACT |
|  | GA2ox8-R | GCGCCAACACCCTGTGCTTCA |
| *GA2ox9* | GA2ox9-F | CGACTTCCTCACCGTGCTCTG |
|  | GA2ox9-R | CGTTCGTCATCACCCTGTGCT |
| *TPS3* | TPS3-F | GGGAAACGATGTCACAACCG |
|  | TPS3-R | TTGCTCTCTCTCATGTGAAGCTATG |
| *TPS20* | TPS20-F | TCCCTGTGGTGCAACGACTTT |
|  | TPS20-R | CCCAACAATCTCCTGAATATACCTG |
| *TPS24* | TPS24-F | CAAAGGGGAAATTGCGAGCACC |
|  | TPS24-R | GCTGATGATGTTCACAACGGGCT |
| LOC_Os02g02930 | 2930-F | TCTCCGCTAGCCCCAGTAGAT |
|  | 2930-R | TCTCCGCTAGCCCCAGTAGAT |
| LOC_Os03g22634 | 22634-F | GGCAGCAAGGGTTATGGGTT |
|  | 22634-R | TGGAGGGCTTCCTCGTTGTG |
| LOC_Os08g07080 | 7080-F | GGGTGATTATATGTACAAGCAAGTT |
|  | 7080-R | ATGTTATACGGCTCCATATAGAGC |
| LOC_Os08g07100 | 7100-F | CTGGGGATTATATATACAAGCAAGCA |
|  | 7100-R | TGCTATATGGCTCAACGTAAAGTGA |
| LOC_Os11g28530 | 28530-F | GCATGGGCTGGTGTTCGGAT |
|  | 28530-R | GTAGCCGTCTCCCTCCTGGTAGA |
| *CYP96B4* | CYP96B4-F | GGGGAGGATGAAGCGGGTGT |
|  | CYP96B4-R | TCTGCACGAGCGCCATCTCC |

**References**

Ruiz-Sola, M.Á., Barja, M.V., Manzano, D., Llorente, B., Schipper, B., Beekwilder, J. and Rodriguez-Concepcion, M. (2016) A single *Arabidopsis* gene encodes two differentially targeted geranylgeranyl diphosphate synthase isoforms. *Plant Physiol.* **172**, 1393-1402.
